# Conducting polymers: a comprehensive review on recent advances in synthesis, properties and applications

**DOI:** 10.1039/d0ra07800j

**Published:** 2021-02-03

**Authors:** Namsheer K, Chandra Sekhar Rout

**Affiliations:** Centre for Nano and Material Sciences, Jain University, Jain Global Campus Jakkasandra, Ramanagaram Bangalore-562112 India r.chandrasekhar@jainuniversity.ac.in csrout@gmail.com

## Abstract

Conducting polymers are extensively studied due to their outstanding properties, including tunable electrical property, optical and high mechanical properties, easy synthesis and effortless fabrication and high environmental stability over conventional inorganic materials. Although conducting polymers have a lot of limitations in their pristine form, hybridization with other materials overcomes these limitations. The synergetic effects of conducting polymer composites give them wide applications in electrical, electronics and optoelectronic fields. An in-depth analysis of composites of conducting polymers with carbonaceous materials, metal oxides, transition metals and transition metal dichalcogenides *etc.* is used to study them effectively. Here in this review we seek to describe the transport models which help to explain the conduction mechanism, relevant synthesis approaches, and physical properties, including electrical, optical and mechanical properties. Recent developments in their applications in the fields of energy storage, photocatalysis, anti-corrosion coatings, biomedical applications and sensing applications are also explained. Structural properties play an important role in the performance of the composites.

## Abstract 2

Conducting polymers are extensively studied due to their outstanding properties, including tunable electrical property, optical and high mechanical properties, easy synthesis and effortless fabrication and high environmental stability over conventional inorganic materials.

## Graphical abstract

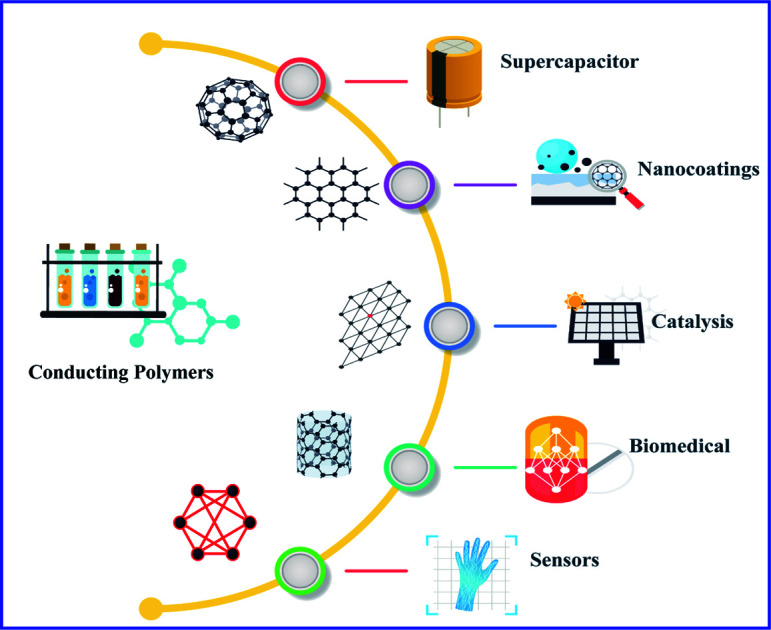

## 1. Introduction

**Fig. 1:**
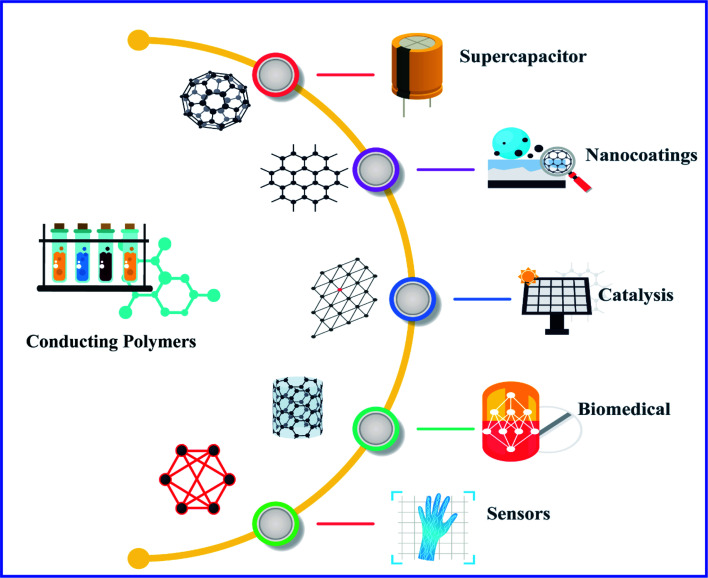
Schematic illustration of applications of conducting polymers and their composites.

**Chart 1:**
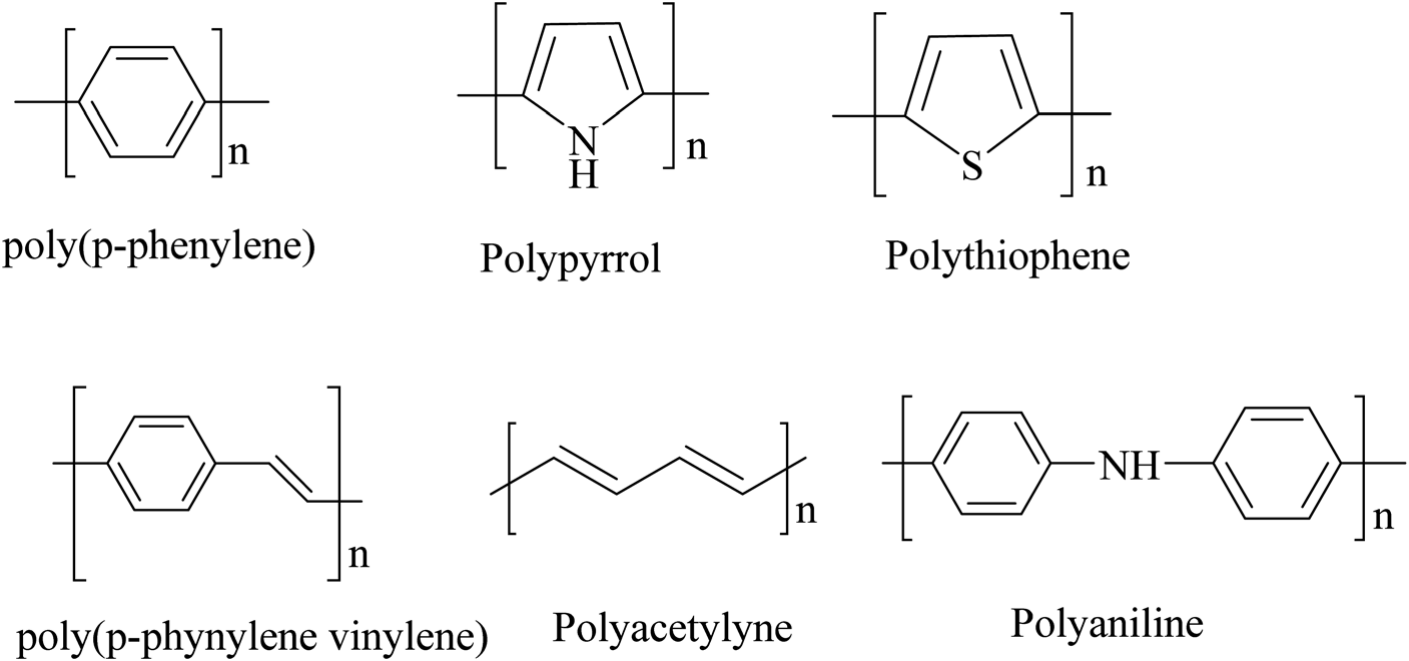
Structural illustration of different conducting polymers.

Polymers were considered to be electrical insulators before the invention of conducting polymers (conjugate polymers), but these organic polymers have unique electrical and optical properties similar to those of inorganic semiconductors.^1^ A conjugated carbon chain consists of alternating single and double bonds, where the highly delocalized, polarized, and electron-dense π bonds are responsible for its electrical and optical behavior. Typical conducting polymers include polyacetylene (PA), polyaniline (PANI), polypyrrole (PPy), polythiophene (PTH), poly(*para*-phenylene) (PPP), poly(phenylenevinylene) (PPV), and polyfuran (PF) (Chart 1). Alan G. MacDiarmid and physicist Alan J. Heeger discovered (SN)_*x*_ sulfur nitride metal, an inorganic material, which showed higher electrical conductivity when doped with bromine, and this finding led to the investigation of conducting polyacetylene. Polyacetylene doped with bromine has a conductivity a million times higher than that of pristine polyacetylene and this investigation was rewarded by a Nobel Prize in 2000.^2^ Conventional polymers consist of thousands to millions of monomer units. They are stiff and soluble in solvents, but a conjugated polymer chain consists of a lower number of monomer units. The mechanical property is gained by the presence of alternating single and double bonds present within it. The solubility and processability of conducting polymers depend mainly upon the attached side chains, and the attached dopant ions give them mechanical, electrical and optical properties.^3^ Conducting polymers are crystalline and partially amorphous. Conducting polymers consist of both localized and delocalized states, and the delocalization of π bonds depends heavily upon disorder, and this delocalization plays an essential role in the generation of charge carriers like polarons, bipolarons, solitons, *etc.*, which are responsible for the transition from insulator to metal.^4^ The conductivity of conjugate polymers acts like an insulator to a semiconductor in their pure form, and the conductivity increases with dopant concentration. In the undoped state, they behave as an anisotropic, quasi-one-dimensional electronic structure with a moderate bandgap of 2–3 eV like a conventional semiconductor and they exhibit the electrical and optical behavior of semiconductors along with the mechanical action of typical polymers. When conjugated polymers undergo doping or photoexcitation, the π bond gets self-localized to undergo nonlinear excitation as polarons, solitons, bipolarons, *etc.*, and the polymer transforms from a nonlinear excitation state to a metallic state.^5–7^ Here in this comprehensive review, we effectively discuss the different synthesis approaches, properties and various applications of conducting polymers (Fig. 1).

## 2. Conducting polymers and their synthesis

### 2.1. Polyacetylene

**Scheme 1:**
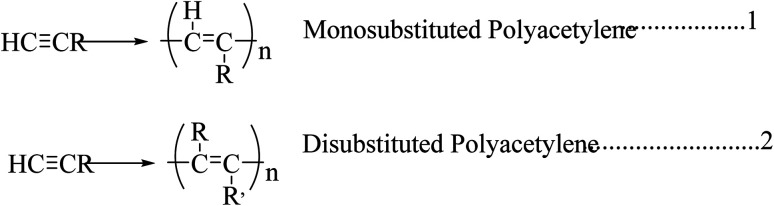
Formation of monosubstituted and disubstituted polyacetylene.

The invention and conductivity enhancement by doping of polyacetylene were rewarded with the Nobel Prize.^8^ Polyacetylene and its derivatives show multifunctional behaviors. On close examination, some of its features can be explored, including electrical conductivity, photoconductivity, liquid crystal properties, and chiral recognition. The main chain of polyacetylene is made up of a linear polyene chain. It is flexible and can be decorated with pendant groups, *i.e.*, the hydrogen molecules present in the alternating carbon can be replaced with a foreign molecule to form a monosubstituted or di-substituted polyacetylene (Scheme 1).^9^

**Fig. 2:**
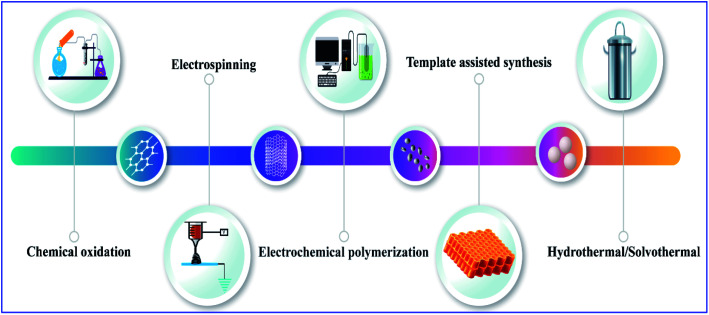
Schematic illustration of different synthesis methods for conducting polymers.

**Scheme 2:**
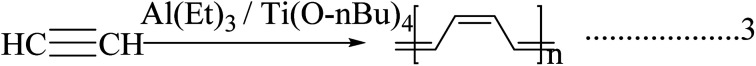
Synthesis of polyacetylene using a Ziegler–Natta catalyst.

Conducting polymers were synthesized using various methods, including chemical oxidation, electrochemical polymerization, vapor phase synthesis, hydrothermal, solvothermal, template-assisted, electrospinning, self-assembly, and photochemical methods, the inclusion method, the solid state method, and plasma polymerization^10–15^ (Fig. 2). Generally, conducting polymers have low electrical conductivity and optical properties in their pristine state; however, doping with suitable materials can give them excellent properties. Polyacetylene has a conductivity in the range of 10^−5^ s cm^−1^, but when the doping level increases, its conductivity rises drastically to 10^2^ to 10^3^ s cm^−1^,^16^ and depending upon the dopant material its properties also change and also give it tunable properties like electrochemical or optical mechanical properties, *etc.*^4,17^ There are several synthesis methods for polyacetylene: catalytic polymerization, non-catalytic polymerization, catalytic polymerization of other polymers, and precursor-assisted synthesis. In the case of the catalytic-polymerization technique, catalysts like Ziegler–Natta catalysts or Luttinger catalysts are used for the synthesis. The polymerization of acetylene produces polyacetylene polymer and oligomers like cyclooctatetraene and vinyl acetylene. There are a lot of catalysts available for the synthesis of polyacetylene and among them Zeigler–Natta catalysts have high solubility in organic solvents and high selectivity. Being a combination of both Ti(0-*n*-C_4_H_9_)_4_ and (C_2_H_5_)_3_A1, they produce highly crystalline free-standing films of polyacetylene on the wall of the reaction flask on which the catalyst is coated (Scheme 2).^18^

**Scheme 3:**
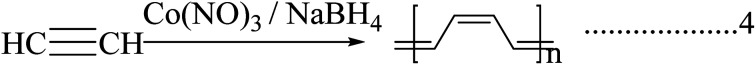
Synthesis of polyacetylene using a Luttinger catalyst.

Luttinger catalysts are also used for the preparation of polyacetylene; they consist of a combination of a hybrid reducing agent and a complex of a group [VIII] metal such as nickel chloride. These catalysts produce high molecular weight polyacetylene without traces of oligomers (Scheme 3). When compared with a Ziegler–Natta catalyst, a Luttinger catalyst uses hydrophilic solvents like water–ethanol tetrahydrofuran (THF) or acetonitrile as a solvent for catalytic action. But it has less catalytic activity than a Zeigler–Natta catalyst and the product formed by these catalysts have almost the same physical and chemical properties.^19^

Electrochemical polymerization of acetylene comes under the heading of non-catalytic polymerization. Anodic oxidation of a monomer precursor in the presence of suitable electrolytes on an inert metal surface is regarded as an electrochemical synthesis. Various electrochemical techniques like cyclic voltammetry, potentiostatic, galvanostatic, and galvanostatic charge discharging techniques have been used for synthesis. The main advantage of this technique is that it will allow the straight deposition of a polymer film on the metal, and we can control the film thickness easily by tuning the electrochemical parameters.^20^ Korshak *et al.* synthesized polyacetylene films by ring-opening polymerization of 1,3,5,7-cyclooctatetraene with a metathesis catalyst, W[OCH(CH_2_Cl)_2_]*n*Cl_6−*n*_(C_2_H_5_)_2_AlCl (*n* = 2 or 3), which is an example of the synthesis of polyacetylene without using an acetylene monomer.^21^ Light-induced synthesis of the conjugated system has also been reported, where irradiation of acetylene gas with UV leads to the production of polyacetylene.^22^

### 2.2. Polyaniline

**Chart 2:**
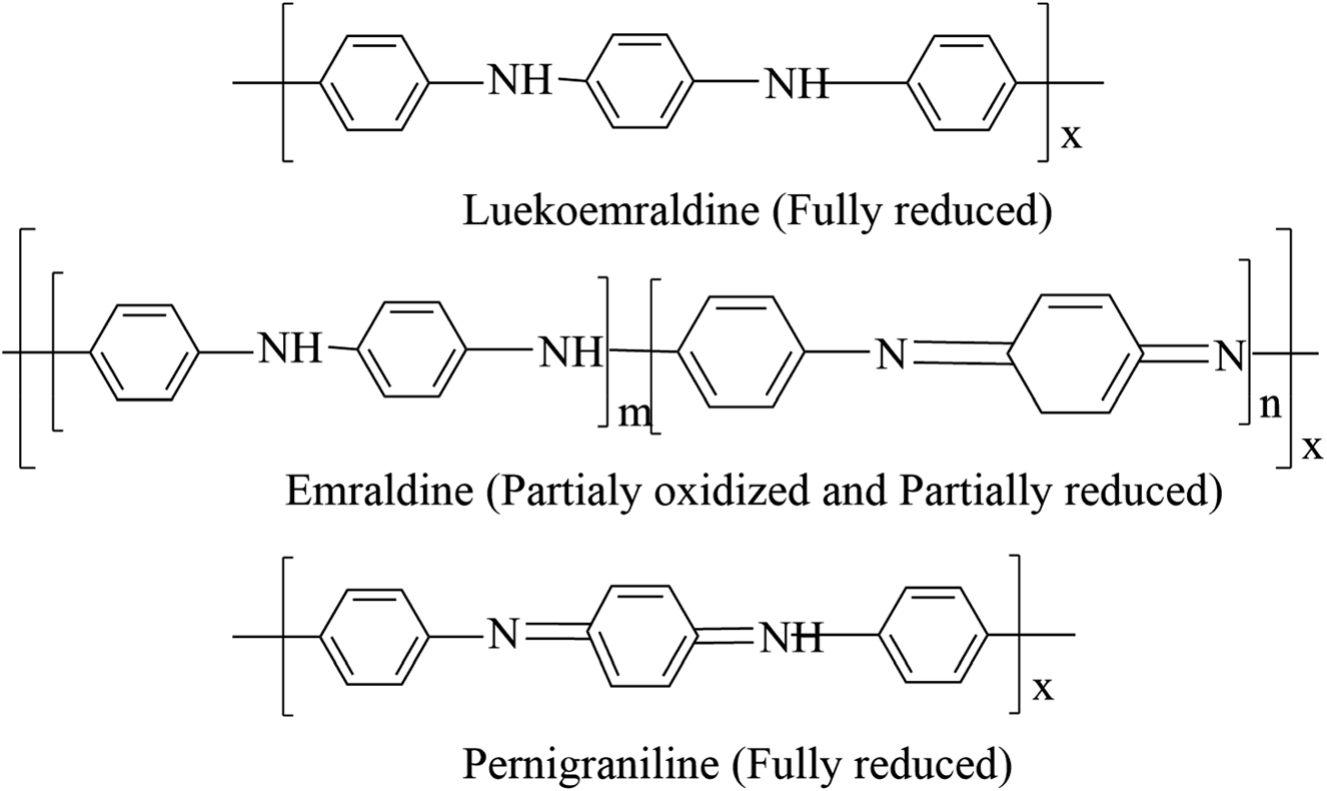
Structural illustration of different forms of polyaniline.

Polyaniline is the most promising and most explored among conducting polymers, and polyaniline has high stability, high processability, tunable conducting and optical properties. The conductivity of polyaniline is dependent upon the dopant concentration, and it gives metal-like conductivity only when the pH is less than 3.^23^ Polyaniline exists in different forms (Chart 2). They are classified as leucoemeraldine, emeraldine, and pernigraniline, by their oxidation state, *i.e.*, leucoemeraldine exists in a sufficiently reduced state, and pernigraniline exists in a fully oxidized state. Polyaniline becomes conductive only when it is in a moderately oxidized state and acts as an insulator in a fully oxidized state.^24^

The polymer backbone consists of both quinoid and benzoid rings, in differing proportions. The difference in the ratio causes the existence of three oxidized states: the fully reduced leucoemeraldine form is in a quinoid state, the fully oxidized pernigraniline form is in a benzoid state and the conductive emeraldine form has an equal ratio of both benzoid and quinoid rings. The dopant does not change its chemical property and will not create any bond with the main chain; it exists in the close vicinity of the polymer chain.^25^

**Scheme 4:**
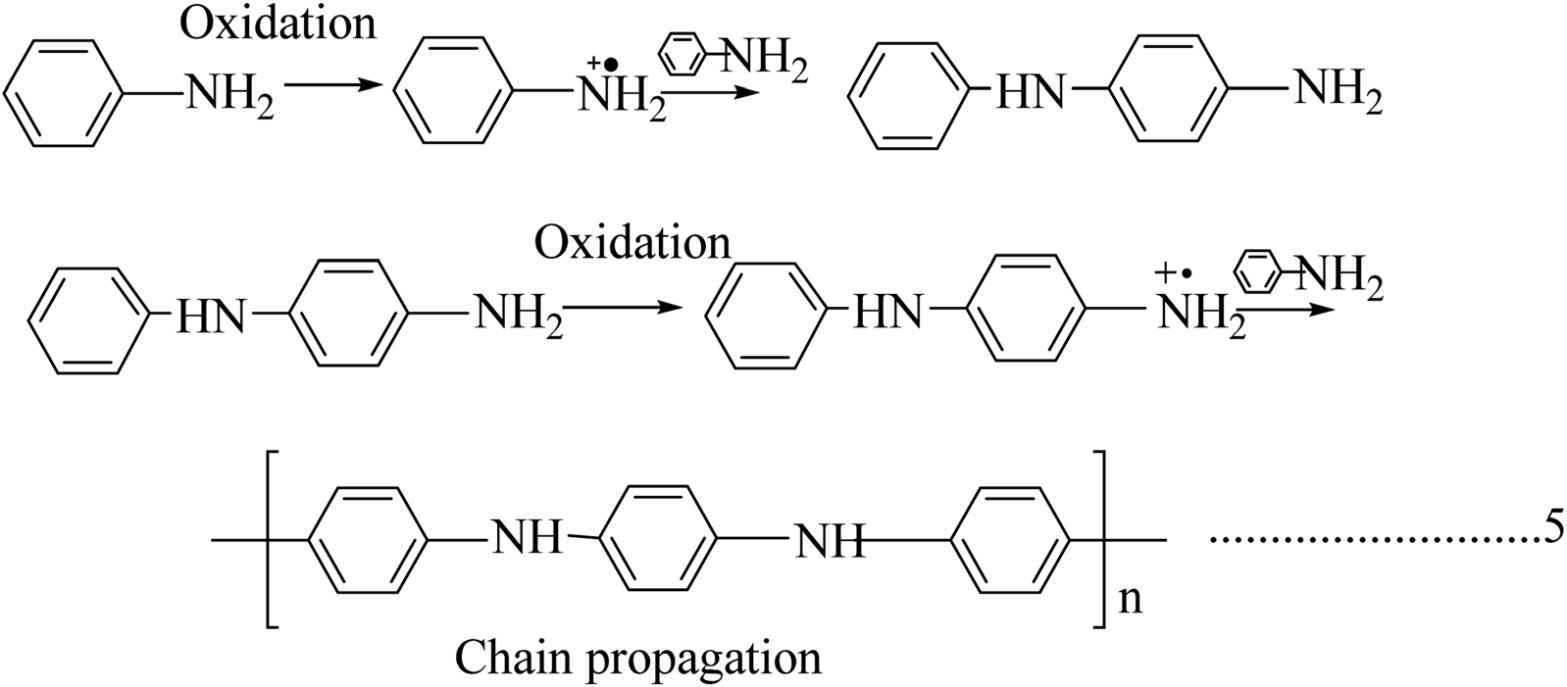
Synthesis of polyaniline by the chemical oxidation method.

The chemical oxidation method is one of the most straightforward methods to synthesize polyaniline; in this method a monomer precursor of the corresponding polymer is mixed with an oxidizing agent in the presence of a suitable acid under ambient conditions to give products, where the doping acid and oxidizing agent are those preferred by the authors concerned (Scheme 4). The change in color of the reaction medium to green indicates the formation of polyaniline. The preparation of the composite also follows the same method. Generally, oxidizing agents like ammonium persulfate, ammonium peroxy disulfate, ceric nitrate, ceric sulfate, potassium bichromate, *etc.* are used. Depending upon the pH of the acid dopant, the conductivity effectively modulates the physical parameters. The polymer and composite possess good conductivity when the pH is between 1 and 3.^26–28^

Interfacial polymerization is also used to synthesize polyaniline, in which an aniline monomer is solubilized in an organic solvent like toluene, an oxidant solution and a dopant acid-containing aqueous solution. Polymerization takes place in the interphase of these two immiscible liquids when an oxidant solution is added to the monomer solution. A microemulsion technique is also followed for the synthesis of polyaniline, where the polymerization also takes place in the interface between two immiscible liquids, but the difference is in the surfactant used.^29–31^ The electropolymerization technique happens without the effect of an oxidant, and is the same in the case of polyacetylene.^32,33^ Electrospinning is also used to synthesise fibrous polymer morphologies of nano or micro diameters under the influence of a strong electrical field. In this case, a high voltage is applied to the polymer droplets, and the charged droplets get stretched due to surface tension, and at a critical point, the liquid erupts and starts to weave on the counter surface. The principles of both electrospraying and electrospinning are the same. Electrospinning is the only method to produce bulk polymer fibrous structure. Conducting polymers and their composites like pure polyaniline, polypyrrole, polyaniline/polyethylene oxide/carbon nanotubes have been prepared by this technique. There are lot of factors dependent on electrospinning, such as the molecular weight of the polymer, viscosity, distance between spinneret and counter surface, temperature, humidity, *etc.*^34–36^

### 2.3. Polypyrrole

Polypyrrole is unique due to its increased commercial interest because of its high stability, enhanced conductivity, and it is relatively easy to form homopolymer and composites from it. Polypyrrole was first prepared by the chemical oxidation of a pyrrole monomer in the presence of hydrogen peroxide, and it is a black powdery material. Polypyrrole behaves like an insulating material in its undoped virgin state, and it shows a constant conductivity of 10^−5^ s m^−1^ when doped with halogenic electron acceptors such as bromine or iodine.^37^ It is not crystalline, and acts as amorphous in nature, but bulk polypyrrole has 15% crystallinity, and the crystalline region is in the monoclinic phase.^38^ For electrochemically synthesized polypyrrole with a thickness of 1 μm and a yellow blackish colour, transformation occurs by an increase in protonation concentration and it has higher stability in air and high thermal stability in the range of 300 °C; thermal degradation can occur due to the loss of dopant anions.^39,40^

**Scheme 5:**
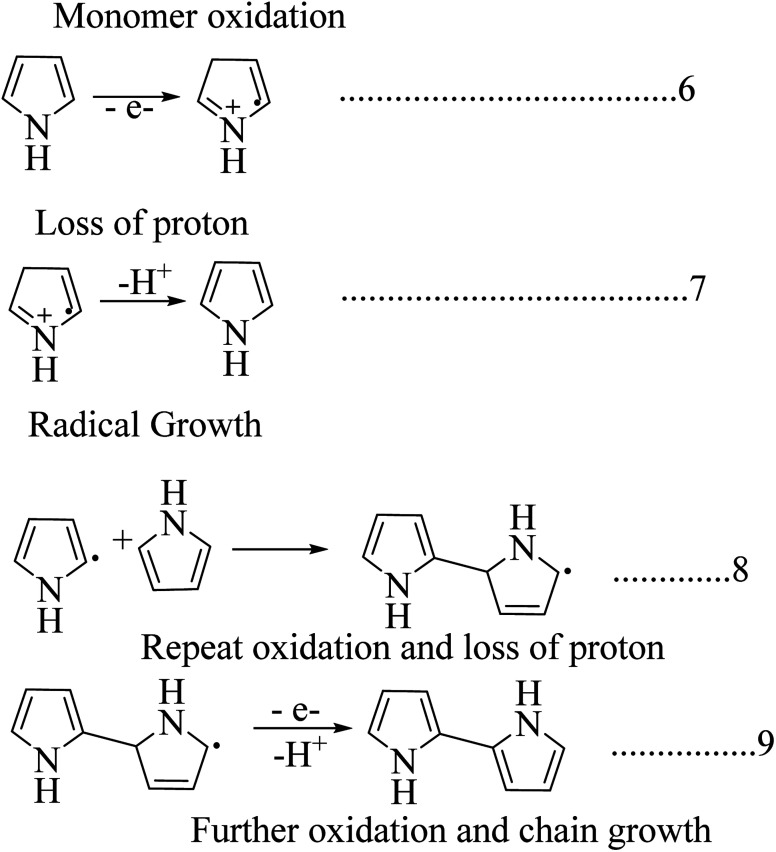
Mechanism of electrochemical synthesis of polypyrrole.

Among the synthesis methods, electrochemical synthesis is widely used to obtain highly conductive polypyrrole, where the procedure and technique are similar to those of other conducting polymers (Scheme 5). The yield of the product is limited in this technique due to the reduced anode size. The main advantage this method has over other techniques is that we can control the thickness and morphology by controlling the electrochemical parameters.^41–44^ There are a lot of electrochemical synthesis mechanisms available. Here in this polymerization mechanism, a free radical cation is developed due to deprotonation, and this free radical attacks the neutral monomer unit. After reoxidation of the dimeric radical and proton loss, the dimeric molecule can experience subsequent oxidation, which results in chain growth. The release of protons in the course of the oxidation of pyrrole was observed experimentally.^45^

Polypyrrole was initially synthesized by the oxidation of a pyrrole monomer in the presence of H_2_O_2_ to obtain an amorphous blackish powdery material.^46^ Pyrrole black is insoluble in organic solvents and shows limited conductivity in the range of 10^−10^ to 10^−11^ s cm^−1^. It is prepared by using acid and peroxide, and its conductivity will increase when doped with a halogen electron acceptor. In general, aqueous or anhydrous FeCl_3_, or other salts of iron(iii) or copper(ii) are widely used as chemical oxidants.^47^

Some factors affect the conductivity and yield: (1) solvent type and oxidant used; (2) pyrrole/oxidant ratio; (3) reaction temperature and reaction time. When polypyrrole is prepared with an FeCl_3_ oxidant, the final product is doped with Cl^−^ anions.

10 *n*C_4_H_5_N + (2 + *y*)*n*FeCl_3_ → [(C_4_H_3_N)_*n*_y+*ny* Cl^−^]^+^ + (2 + *y*)*n*FeCl_2_ + 2*n* HCl

Highly conductive polypyrrole is synthesized by controlling the oxidation potential of an aqueous solution by adding an oxidant to it. Apart from metallic salts, polypyrrole has been synthesized using a halogen electron acceptor, such as bromine or iodine, in various solvents.^37^

### 2.4. Poly(*p*-phenylene)s

Poly(*p*-phenylene) is a macromolecule made up of benzoid aromatic nuclei, directly joined by C–C bonds. Poly(*p*-phenylene)s attract considerable interest due to their high thermal stability, high air stability, and ease of doping and tunable conducting and high optical properties. The mechanical rigidity of the polymer backbone along with conjugation is a promising development in the field of nonlinear optics. The solubility of poly(*p*-phenylene)s is limited but gradually increases when flexible side chains are attached to the backbone. The conductivity of poly(*p*-phenylene) will grow 14 times when it is doped with suitable dopants, and it allows both p-type and n-type doping. The conductivity increases with dopant exposure time.^48^ Poly(*p*-phenylene)s play a vital role in the fabrication of organic LEDs due to their improved optical property and blue light emission compared to other conjugated systems. The structural features of poly(*p*-phenylene) are dependent on temperature: it shows a planar structure at higher temperature ranges, and it shows phase transition and a distorted planar structure at lower temperature ranges. Poly(*p*-phenylene) has a tunable optical band gap, and structural changes occur with the addition of suitable dopants or by adding side chains.^49^ Poly(*p*-phenylene) exhibits higher mechanical properties; also, it shows very high tensile properties compared with engineering polymers. It shows a two times higher modulus and strength compared with polyetheretherketone (PEEK) or thermosetting polyimide (PI). Polyparaphenylene has various uses in various mechanical applications within the temperature range of 140 °C.^50^

**Scheme 6:**
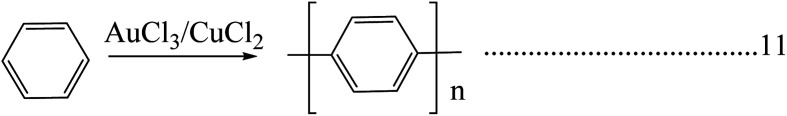
Synthesis of poly(*p*-phenylene)s using a binary system (both a Lewis acid and an oxidant system).

**Scheme 7:**
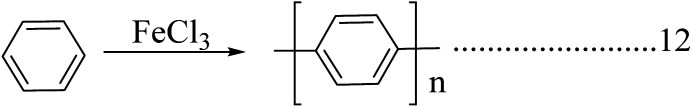
Synthesis of poly(*p*-phenylene)s using single system (oxidant system).

Direct oxidation of benzene molecules is widely using for the synthesis of poly(*p*-phenylene)s. Here in this procedure dehydro coupling of benzene nuclei with an oxidative catalyst leads to the formation of carbon–carbon bonds. The polymerization reaction is carried out by using reagent consisting of a binary or a single system (Schemes 6 & 7). The binary system consists of both a Lewis acid and an oxidant system, and in the case of a single reagent system (FeCl_3_), the system acts as both a Lewis acid and an oxidative system by itself. A combination of an AuCl_3_ and CuCl_2_ system is an example of a binary reagent system, where AuCl_3_ acts as Lewis acid, and CuCl_2_ acts as an oxidant. Reactions are carried out at temperatures of 36–37 °C.

**Scheme 8:**
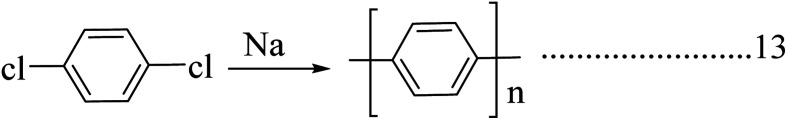
Synthesis of poly(*p*-phenylene)s using a Wurtz–Fittig reaction.

**Scheme 9:**
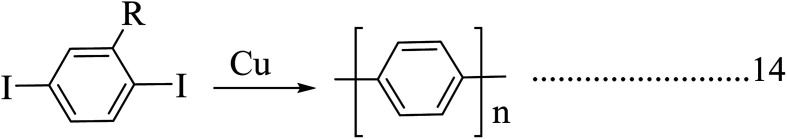
Synthesis of poly(*p*-phenylene)s using an Ulman reaction.

The first chemically synthesized poly(*p*-phenylene) was obtained using a Wurtz–Fittig reaction, which is a metal coupling reaction (Scheme 8).^51^ The Ulman reaction is also used for getting poly(*p*-phenylene)s and the products obtained by this method have lower molecular weight and fewer structural irregularities (Scheme 9). But this method is useful for the preparation of substituted phenyls such as methyl and nitro groups.^52^

The precursor method was also investigated for the synthesis of poly(*p*-phenylene)s. Conducting polymers are insoluble in nature and the preparation of the targeted polymer from an insoluble precursor polymer is an excellent research topic. The Marvels, Grubbs, and ICI precursor method was famous for the production of poly(*p*-phenylene)s. In the case of the Marvels method, the main drawback is that the product will have low molecular weight and decreased stereochemical control.^53^ Electrochemical synthesis and reductive polymerization have also been used.^37^

### 2.5. Poly(*p*-phenylene vinylene)

Poly(*p*-phenylene vinylene) was the first electroluminescent material used for the fabrication of organic light-emitting diodes due to its high optical property. It was extensively studied for the fabrication of LED displays. The crystallographic studies of poly(*p*-phenylene vinylene) revealed its amorphous nature and, later, the isotropic distribution of crystallites with monoclinic unit cells was observed.^54,55^ In the case of poly(*p*-phenylene vinylene), the crystallite distribution was found to be in two planes. The electrical properties of the poly(*p*-phenylene vinylene) change in some orders is dependent on dopants, and it behaves as an insulator in its pristine form. The electrical properties of pristine poly(*p*-phenylene vinylene) depend upon the structural behaviour and also depend on the reaction conditions. When doped, its conductivity value changes from 10^−13^ to 10^3^ s cm^−1^.^56^ Poly(*p*-phenylene vinylene) has potential applications in optoelectronic fields like LEDs, lasers, photodetectors, *etc.* Due to its improved mechanical property it was used in the area of LED panels and the first fabricated poly(*p*-phenylene vinylene) panel was structured by sandwiching poly(*p*-phenylene vinylene) between an ITO anode and a low work function metal cathode.^57^

**Scheme 10:**
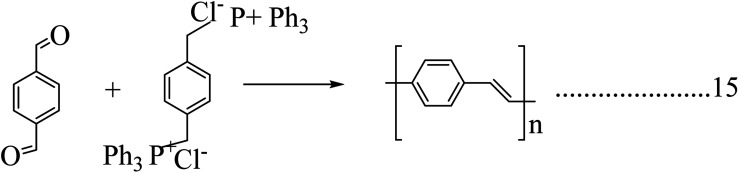
Synthesis of poly(*p*-phenylene vinylene) by a Wittig coupling reaction.

There are a lot of methods available for the preparation of poly(*p*-phenylene vinylene). The Wittig coupling reaction has been studied extensively. In this reaction procedure, the coupling between an aromatic bisphosphonium salt and a bisaldehyde yields poly(*p*-phenylene vinylene) (Scheme 10). A Suzuki coupling reaction was also investigated for the production of poly(*p*-phenylene vinylene) by Pd-catalyzed coupling of alkyl-substituted aryldiboron acids with dibromo aromatic compounds.

Other synthesis methods have also been reported, such as electropolymerization,^58^ benzoine condensation, ring-opening polymerization, metathesis polymerization, and chemical vapor deposition.^37^

### 2.6. Polythiophenes

Polythiophene and its derivatives are extensively studied for their environmental stability, thermal stability and high optical property compared with other conducting polymers. Polythiophenes are widely used to fabricate non-linear optical devices, photochromic modules, polymer LEDs, anticorrosion coatings and are used in energy storage devices. The electronic and optical properties of polythiophenes can be modulated by doping engineering or by chemical modifications. The band gap of polythiophenes varies 3–1 eV depending on the dopant and side chain employed.^59^ Poly(3,4-ethylenedioxythiophene) (PEDOT) is an important derivative of polythiophene and it was thoroughly studied for its high electrical and electro-optical properties. The main problem with the PEDOT derivative is its insolubility in water. This was successfully overcome by introducing a polyelectrolyte like polysulfonates (PSS) into the PEDOT matrix. PSS act as both a dopant and a stabilizer by a charge balance mechanism. The PEDOT:PSS derivative has high conductivity, good mechanical flexibility and long-term thermal stability.^60^ The electrical properties of polythiophene and its derivatives are enhanced through solvent treatment, the introduction of a surfactant and by varying the PSS concentration. Poly(3-hexylthiophene) (P3HT) is another class of derivatives of polythiophenes and their applications are mainly focused on the opto-electronic and electronic fields. P3HT is popular because of its wide availability, low cost, well-known morphology and easy processability. P3HT is a semicrystalline polymer and its backbone is made up of isolated rings and linear side chains. This structural arrangement enables the freedom to sample conformational space. The glass transition temperature of P3HT is recorded as 12 °C and it has a high tensile modulus of 200 MPa to 1 GPa, which varies in accordance with the synthesis method and the purity of the sample.^61^

**Scheme 11:**
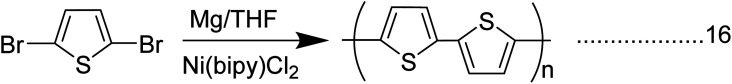
Synthesis of polythiophenes by the Yomomoto route.

**Scheme 12:**
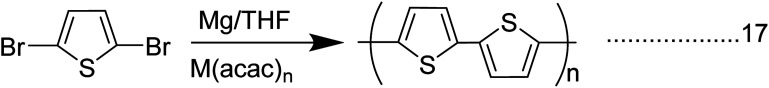
Synthesis of polythiophenes by the Lin–Dudek route.

Polythiophene was chemically synthesized in the early 1980s by the Yamamoto and Lin–Dudek routes (Schemes 11 & 12). Other advanced techniques, like direct sol–gel, oxidative synthesis,^62^ organometallic coupling reaction,^63^ electropolymerization, template-assisted synthesis, and hydrothermal and solvothermal techniques, have been effectively studied.^60^

For the synthesis of polythiophene derivatives like PEDOT, PEDOT:PSS and P3TH, the composite was achieved using various techniques, including green synthesis,^64^ synthesis in microfluid systems, electropolymerization and by some other novel techniques.^65,66^

## 3. Electrical and electronic properties

The electrical properties of a material are usually explained using its electronic band structures. The energy difference between the conduction band and the valance band classifies materials from insulators to conductors.^67^ Intrinsically conducting materials have a decreased bandgap, and the conduction and valance bands overlap. The electronic band theory clearly explained the case of conducting polymers, but some other studies have also revealed the transport properties of conducting polymers rather than band theory. All conducting polymers have conjugate bonds in their backbones, and these bonds are responsible for the movement of electrons: *i.e.*, a single bond contains a localized σ bond and a double bond has both σ and weaker π bonds. The dual relationship between first and second carbons includes a π bond and this π bond transfers to the second and third carbons, and the π bond between the third and fourth carbon transfers to the next pair; this displacement of π bonds allows the electrons to flow.^4^ The conductivity shows drastic changes depending upon the dopant material, the arrangement of the polymer chain, and its length. The dopant concentration and pH value enhance the conductivity; for example, polyaniline shows excellent conductivity if the pH is maintained between 0 and 3.

**Fig. 3:**
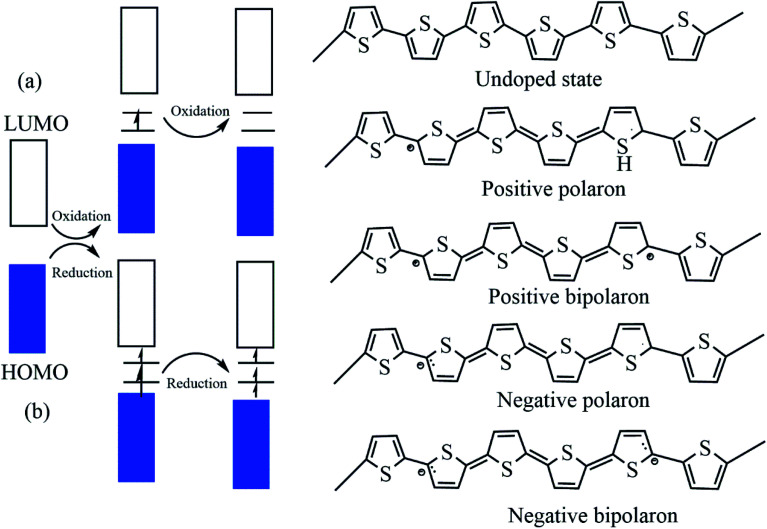
The electronic band and chemical structures of polythiophene (PT) with (a) p-type doping and (b) n-type doping (DOI: 10.3390/polym9040150, Open source MDPI).

Undoped pristine polymers may act as an insulators or semiconductor, and conductivity increases with increasing dopant concentration. The dopants exhibit redox reactions, and they add and withdraw electrons from the conducting polymers.^68^ There are two types of doping: p-type doping and n-type doping, and the dopants produce both positive and negative polarons/bipolarons. These charge carriers are delocalized over the polymer chains, which facilitates the electronic conductivity.^16,69^ In the case of p-type doping the electrons migrate from the HOMO of the polymer backbone to the dopant species and a hole is created, and this hole creates a deficiency of electrons in the backbone. But in the case of n-type doping the electrons transfer to the LUMO of the polymer backbone and electron density occurs, and these charge carriers enhance the conductivity (Fig. 3). There is academic interest in p-type doping because of the high stability of positively charged carriers.^70–72^

**Fig. 4:**
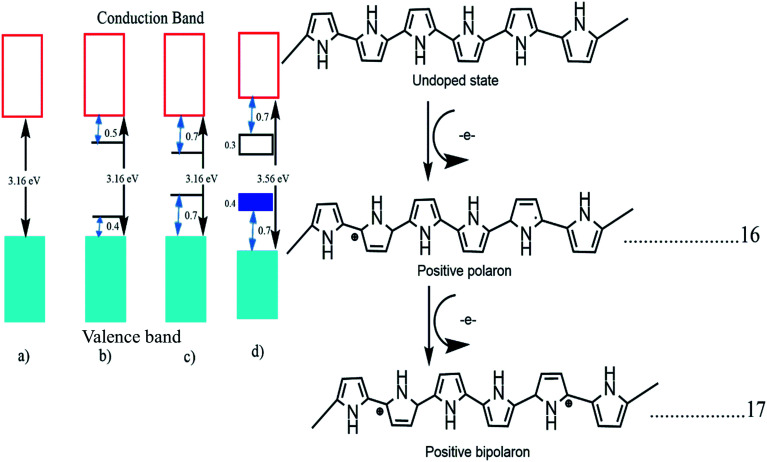
Electronic bands and chemical structures illustrating (a) undoped; (b) polaron; (c) bipolaron; and (d) fully doped states of polypyrrole (PPy) (DOI: 10.3390/polym9040150, Open source MDPI).

The band structure of polypyrrole with different stages of doping was tested. Pristine undoped polypyrrole acts as a semiconductor material with a high bandgap of 3.16 eV and when it is doped with p-type materials the polymer backbone gets oxidized, and the loss of π electrons from the polymer backbone occurs. This electron loss deforms the polymer structure from benzoid to quinoid and generates one polaron in the backbone. This polaron creates a localized electronic level within the band structure, and further oxidation of bipolarons occurs due to the removal of π electrons.^73^ This transformation from benzoid to quinoid form is faster in the case of bipolarons. If it is oxidized, again the bipolarons overlap, and a separate small bipolaronic band is created within the main band structure, and the band energy is reduced to 1.4 eV from 3.16 eV. In Fig. 4, we can see the transformation of a semiconducting polypyrrole to a metallic polypyrrole.^47^

The unit cell present in the polymer backbone interacts with the neighboring unit and creates a valance band and a conduction band. Conjugative polymers have high energy levels while ionizing, and once the polymer becomes ionized, transformation of the band structure occurs. The change may be from benzoid to quinoid.^74^

**Fig. 5:**
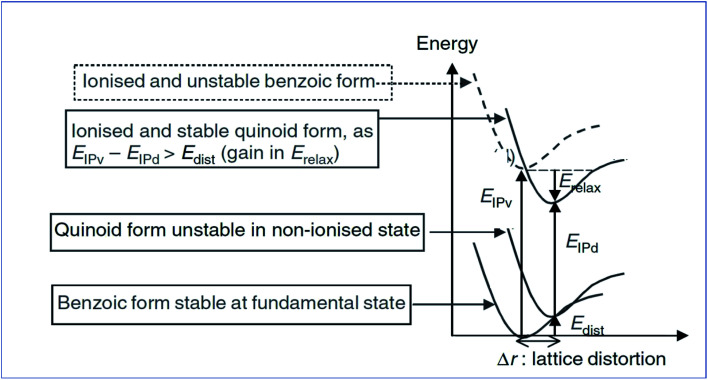
Energy level diagram of the molecular ionization process of poly(*p*-phenylene) (reprinted with permission,^63^ copyright 2004 Wiley).

Analyzing Fig. 5 shows that *E*_ip-v_ is the vertical relaxation energy, and *E*_rel_ is termed the relaxation energy gained while ionizing, and *E*_dis_ and *E*_ip-d_ are the distorted energy in the ground state and ionization energy of the distorted molecule, respectively. The distortion energy leads to an upward shift of the HOMO and a downward shift of the LUMO. For the conducting polymer polyacetylene, the valance band is filled by sp^2^ orbitals of the carbon atom and the s orbital of the hydrogen atom, but the conduction band is empty. The bandgap energy is almost 10 eV, and it is too high.^75,76^*Trans*-polyacetylene has the novelty of exhibiting a degenerated energy state, *i.e.*, it has two geometrical structures with the same energy, and the difference in geometry arises due to the exchange of a single bond between carbon–carbon interactions. The actual conducting mechanism in polyacetylene is well understood. As discussed above, polyacetylene acts as an insulator in its pristine form and after doping with electron-deficient or electron-withdrawing dopants (AsF_3_, Br_2_,i_2_/CCl_4_) it forms radical cations or polarons by removing electrons from the π bond, because the removal of an electron from the π bond is relatively easy compared to removal from a σ bond. Then after treating with dopants again, it generates bipolarons by taking one more electron from the radical present in the polaron. After the generation of bipolarons, the π bonds are shifted in between σ bonds to form solitons. The solitons can be negative or positive depending upon the dopant used.^77^

**Fig. 6:**
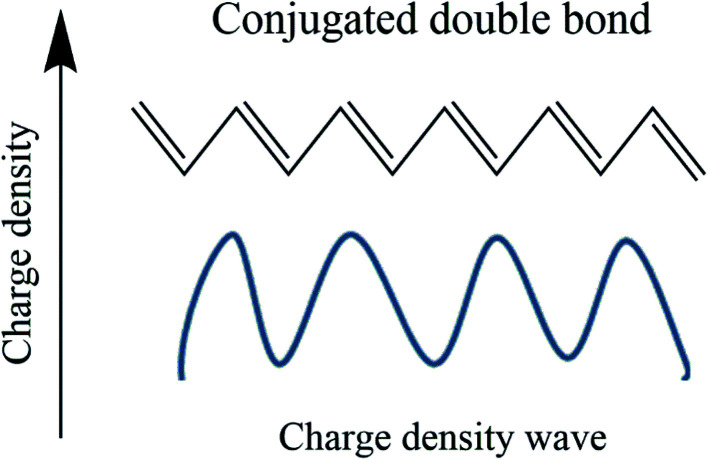
Representation of charge density wave in polyacetylene.

The conduction mechanism in conducting polymers has two sides. One is a chemical aspect, and the other is the solid-state physics aspect. Chemists approach a conducting polymer as a conjugative bond system, while physicists see it as a charge density wave,^78^ as shown in Fig. 6. In the case of double bonds which are more electron dense than σ bonds, the movement of a π electron is related to the oscillation of a charged density wave, and more specifically it is a bond order wave because electron density is not confined at the lattice site.^79^

### 3.1. Charge carrier transport models

In the conduction mechanism of conjugate polymers the charge carriers are responsible for the conduction. In polypyrrole, bipolarons trigger the conductivity while in *trans*-polyacetylene, solitons act as charge carriers. In conducting polymers, the charge carriers hop not only in the defective area but also in interchain gaps. Mott explains that electrical conductivity depends on temperature and concentration. Temperature dependence on conductivity can be explained by some models.^80^

#### 3.1.1 Mott model

The Mott model has been effectively used to explain the conduction mechanism in amorphous materials and organic polymers, and this model tried to explore the role of temperature on conductivity.^81^ In this model, Mott attempted to explain the temperature behaviour of distorted systems, and he observed that charge carriers move from one localized energy state to another localized energy state with different energy, through the absorption of phonon energy. There are main two factors that the hopping (the movement of an electron from one localized energy state to another) mechanism depends on: the tunnelling factor 
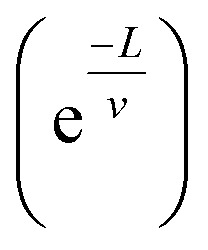
 where *v* is the localization length and *L* is the hopping distance) and the Boltzmann factor.^82^i
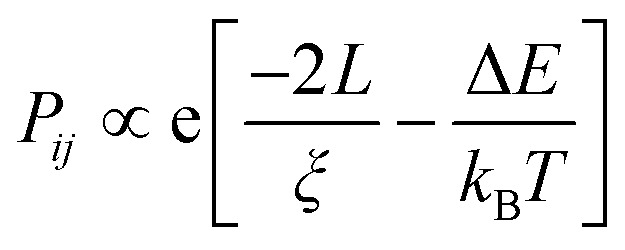


*L* and Δ*E* have a connection with the density of states *N*(*E*_F_).

*L* ^*d*^Δ*E N*(*E*_F_) ∼1 where *d* is the dimensionality of the system. From the above equation, it is clear that the product of the number of available states in a volume *L*^*d*^ energy interval and the density of states is of the order of one.ii
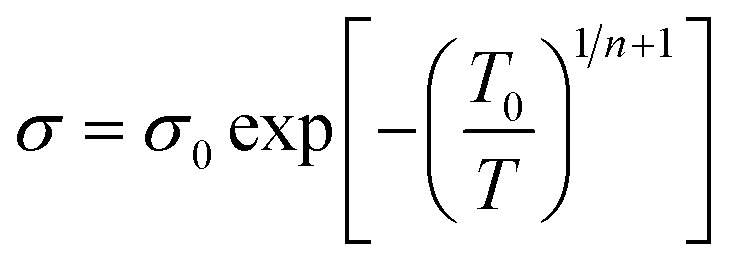
here *n* is the dimensionality of charge transfer, *σ*_0_ is conductivity at room temperature and *T*_0_ is Mott's temperature constant:iii
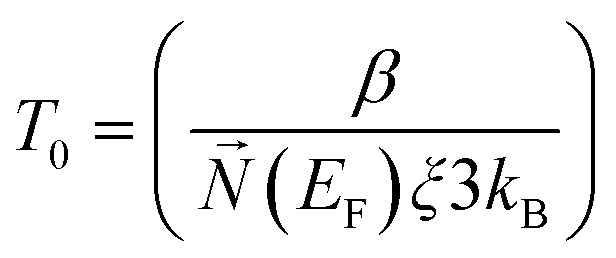


Investigation of the Mott model is done by plotting a graph of log *σ vs. T^−γ^*, where the least square fitted curve gives an idea about both dimensionality and the charge transport mechanism.

#### 3.1.2 Schaefer–Siebert–Roth model

This model is slightly different from Mott's model: in the Mott model localization length and hopping distance are taken into account, but here localization length with conjugation length are taken into account. When polyacetylene is doped with p dopants or n dopants, breakage of a π bond and charge carriers like polarons and bipolarons occur, and the formation of these charge mobilizers causes the formation of different conjugation lengths within the backbone.^83^iv
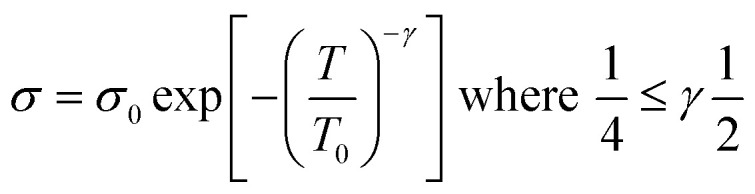
here *σ*_0_ and *T*_0_ depend upon localization length, and *γ* depends upon the density of states at the Fermi level.

## 4. Optical property

The electronic structure of conjugated polymers is anisotropic and quasi-one-dimensional due to the presence of π bonds in the polymer backbone by utilizing electron–phonon interactions. The electronic transport behavior in organic semiconductors is usually due to the influence of charge mobilizers like solitons, polarons, or bipolarons in the ground state degeneracy. The sub-gap optical transitions occur in the polymer backbone while doping triggers charge mobility by a shift of oscillator strength from π to π*. These nonlinear excitations are responsible for the charge mobility. The conjugated polymers behave like semiconductors in their pristine form, and they act metallically when doped with p and n dopants. In the nonlinear excitation of conjugated polymers,^84^ there are some conflicts with the photoexcitation of conjugated polymers, *i.e.*, polarons or bound neutral excitons.^85,86^ Before understanding the optical property of conjugate polymers, we need to know the basics of the physical properties of simple solids. The optical constants of solids give a complete idea of both vibronic and electronic properties, when an electromagnetic wave interacts with the polymer. The response of the system is characterized by the function of dielectric constants.^87^v
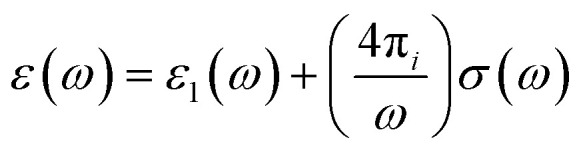
*ε*_1_(*ω*) and *σ*(*ω*) are the real part of the dielectric function and frequency-dependent conductivity. A reliable way to determine the optical property of a solid is to shine a monochromatic light onto the sample and calculate the values of reflectance and transmittance as a function of phonon energy. The reflectance data gives an idea of electronic structure, optical conductivity, and dielectric constant after analyzing the reflectance spectra using Kramers–Kronig analysis.^88^

### 4.1. Electroluminescence, electrochromic and photochromic effects of conjugated polymers

**Fig. 7:**
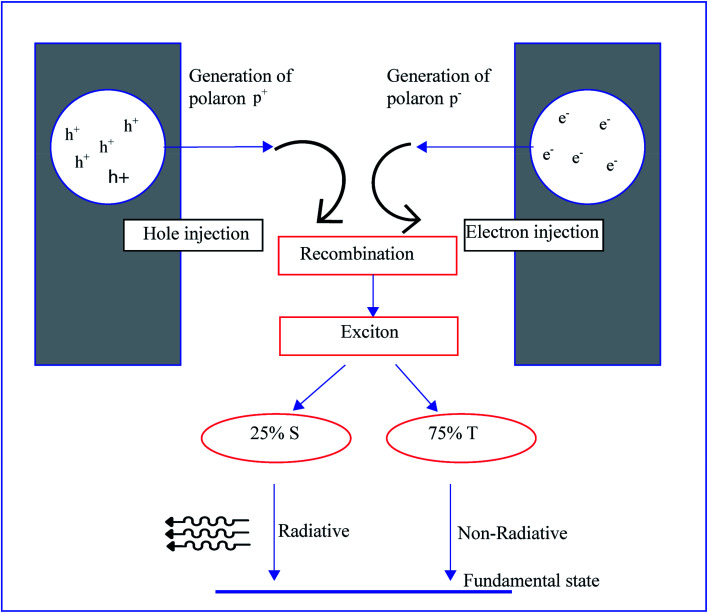
Schematic illustration of electroluminescent mechanism of conducting polymer diodes.

Electroluminescence is a tendency to generate light by electrical excitation, and its typical behavior is observed in both organic and inorganic semiconductors. As we can see in Fig. 7, electroluminescence requires the pumping of both electrons and holes from respective electrodes, and these opposite charges are recombined to form excitons. The radioactive decay of excitons occurs by this recombination.^89^ Electroluminescence in an organic film was first investigated in 1980 by using a two-layer sublimed molecular device. This device was constructed by using two layers of thin films consisting of a hole transporting layer made up of aromatic diamine and an electron transporting layer of 8-hydroxyquinoline aluminum. Indium tin oxide and magnesium silver alloy were used as hole injectors and electron injectors, respectively.^90^ A wide variety of organic polymers are used as transport layers. The conjugated polymers show semiconductor to metallic behavior by the movement of π bonds present in the backbone. The delocalization of π bonds generates charge mobilizers like polarons, bipolarons, solitons, *etc.* The electroluminescence in conducting polymers was first reported in poly(*p*-phenylene vinylene). The energy band gap of poly(*p*-phenylene vinylene) is around 2.5 eV, and this value lies between π and π*, and poly(*p*-phenylene vinylene) generates green-yellow luminescence of energy lower than its energy gap.^57^ Generally, a molecule contains a single and a double bond. The π electron present in the double bond is excited to a higher energy state by absorbing photon energy which is more significant than the band gap. Similarly, in the case of conjugated polymers, the π electron present in the alternating double bonds on the backbone transfers from the HOMO to the LUMO and the energy gap *E*_g_ can be even smaller. When the polymer absorbs photon energy, electron transfer from the HOMO to the LUMO occurs and the electron–hole pairs generate excitons. The conducting polymers exhibit phosphorescence and fluorescence. If the spins of both excited and lower states are the same, that kind of light emission is called phosphorescence; if spins are opposite the emission is called fluorescence. Conducting polymers show a novel optical property when doped with fullerene and the disubstituted polyacetylene shows higher photoluminescence quantum efficiency than in an undoped or monosubstituted state.^91^

Optical changes in conducting polymers occur during chemical reactions and by the effect of external factors like a strain effect on the polymer matrix as well as a shift in planarity. There are a lot of physical and chemical elements influencing the optical property like excitation and ionization impurities, phonons present in the polymer, recombination, excitons, strain factor, *etc.* The degree of anisotropy, conjugation length and topochemical reaction are three specific chemical aspects influencing the optical property.^92^

Topochemical reactions are triggered by exposure to UV radiation, gamma radiation and X-ray radiation, by thermal annealing. Solvent types also induce topochemical reactions. The criteria for this kind of reaction are that the stereochemistry of the dimer is determined by the contact geometry of the nearest neighbor's double bonds, and the distance between adjacent monomers must be less than 4 Å.^93,94^ A topochemical reaction is used to conduct polymerization of a conjugate system such as polydiacetylene or polyaniline. Irradiation causes hydrogen bonding between polymer and head groups. This weak intermolecular hydrogen bond bends the polymer backbone and builds it into a zigzag structure which causes a change in color to red. A strong intermolecular bond makes an extended chain-type structure that gives a blue color to polyacetylene. The temperature also affects the optical behavior, *i.e.*, polydiacetylene shows a red-to-blue transformation at low temperatures, and poly(3-hexylthiophene-2,5-diyl) (P3HT) changes from yellow to magenta at higher temperature.^95^ The same trend was observed in polymers when changing the solvent ratio and conjugation length. Conjugation length is defined as the number of uninterrupted π bonds in the conjugated system. On the other hand, it is the defined length of the oligomer containing a specific number of monomer units. Samuel *et al.* developed a model that relates molecular weight to conjugation length with absorbance. When polymers have higher conjugation length, the polymer will show high molecular weight due to the presence of the π bond because a high number of π bonds increases the molecular weight, which leads to a higher absorbance rate.^96^

Photochromism is a phenomenon of light-induced reversible change in the color of chemical species. The two forms have different absorption spectra. The change occurs with a change in physical properties, including viscosity, dielectric constants, surface wettability, and refractive index. This controlled modulation of properties paves a path to the field of optical switches. The light-induced transformation enables a transition from thermodynamically stable state A to less stable state B, in which both states have different absorption spectra. The inter-transition also allows movement from B to A either thermally or photochemically. Generally, most photochromic materials exhibit a colorless state when they are stable and a color transition occurs when moving to an unstable state. Both inorganic and organic materials exhibit a photochromic effect.^97^ Organic materials like polyaniline, polypyrrole, PEDOT, PEDOT:PSS, and P3TH show a photochromic effect due to the pericyclic reaction, *cis*–*trans* isomerization or intramolecular hydrogen/group transfer process.^98^ Organic field emitting transistors have a huge global market and research interest due to their wide applications in the fields of flexible devices, memory, sensors, and smart cards. The fabrication of conventional OFETs with functional molecular blocks is the latest research trend due to the sensitivity of organic functional groups towards pressure, temperature, light, *etc.*^99^ The change in color with changes in viscosity, wettability, refractive index, and solubility in photochromic materials have applications in sophisticated electronic devices. Photochromic camera lenses and photochromic camera filters led the global market and were replaced by organic conducting polymers due to their low weight and higher comfort.

On the other hand, most conducting polymers show electrochromic properties. Electrochromism is defined as an external voltage triggering a reversible change in the optical property of a material.^100^ The first electrochromic device was fabricated by Deb *et al.* during research into amorphous and crystalline materials. Transition metal oxides were the most studied and explored in the field of electrochromic materials and were later followed by small organic materials. Conjugated polymers, another set of electrochromic materials, are in the limelight due to their high optical contrast and easy modification of their structures to develop new electrochromic devices.^101^ Generally, electrochromic materials are classified based on their electronically accessible optical states. The first set of materials like PEDOT have only one colored state during the application of an external voltage. The second type of materials including polythiophenes have two colored states and the third class of materials have more than two colored states depending on their redox state.^102^ Some conducting polymers like polyaniline^103^ and poly(3,4-ethylene dioxythiophene)^104,105^ are inherently multi-colored systems and have a wide variety of optoelectronic applications. The mechanism behind electrochromism is a change in pi-electron character with electrochemical oxidation or reduction. Electrochromic cells,^106^ LCDs and electrochromic smart widows are the main applications of electrochromic materials.^107^

## 5. Mechanical properties

**Fig. 8:**
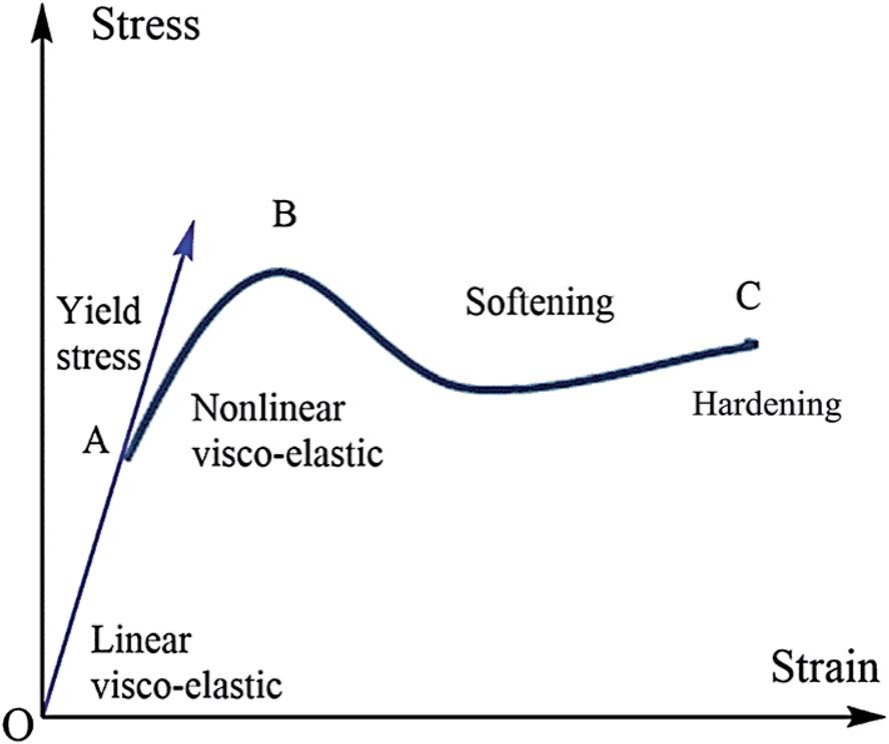
Typical stress–strain curve of polymeric materials.

The mechanical properties of polymer materials depend upon the monomer arrangement and crystallinity. A crystalline polymer has better mechanical properties compared with amorphous semi-crystalline polymers. The macroscopic mechanical property of conducting polymers depends upon the microscopic change in the molecular mobility of macromolecules. Molecular mobility depends upon factors like the structure of branching polymer conformations and macroscopic properties like pressure–temperature *etc.* In the case of amorphous polymers, the distribution and arrangement of monomers are random, and crystalline polymers are not stacked. The molecular motion is higher in amorphous polymers, and when temperature reaches *T*_g_ the polymer is transformed to a rubbery state from its glassy state. This transition leads to a change in mechanical properties. The mechanical property of a polymer depends heavily upon the molecular weight: *i.e.*, the toughness and strength parameters are related to molecular weight and chain entanglement.^108–110^ The engineering stress–strain curve of the polymers is a plot between strain on the *x*-axis and stress on the *y*-axis (Fig. 8). A graphical model of the stress–strain curve is given below. From the graph of the region, OA is represented as an elastic region. In the elastic region, the material will return to its original place after removing the load. The region AB represents the plastic region. The movement of the molecule is high, and deformation occurs on the material when the load is removed. In the elastic region, the material obeys Hook's law. After point A (the elastic limit), the material becomes visco-plastic and permanent deformation occurs.^111^

### 5.1. Elasticity and Young's modulus

The conducting polymers both expand and contract electrochemically: *i.e.*, a redox reaction causes the elasticity. When anions are excluded from or included in the polymer matrix, it becomes strained. The magnitude of the strain depends upon the number of anions. The electrochemical deformations are utilized in the application of artificial muscles. When the polymer is oxidized by applying a positive voltage with a suitable electrolyte, the material loses electrons from the polymer and a pair of anions is formed in the electrolyte. This anions cause the expansion of the polymer. The contraction with reduction is similar to expansion with oxidation. Investigation of stress–strain analysis has been undertaken using different techniques like AFM, nanoindentation, a conventional pull test, and electrochemomechanical actuators.^110^

The mechanical property depends upon the molecular weight of the polymer, and the effect of different molecular weights *M*_n_ = 20, 25, 90, and 110 kg mol^−1^ on the modulus of poly(3-hexylthiophene) has been investigated. The results reveal that a material with a lower molecular weight shows brittle tensile behavior, constrained molecular mobility, and the extended chain crystals cannot withstand a higher strain rate. When the molecular weight increases, the moduli increase drastically, and they provide a high elongation of 300%. Another procedure is to place the film over a water surface, where the same trend is observed. A material with a higher amount of ductility and toughness shows resistance against fracture, which helps for stretching over curved surfaces like vehicle body parts. A comparison of the moduli in both techniques of film on water and film on an elastomer was undertaken. From these two tests it is observed that the modulus value is three to six times higher in the case of the film on an elastomer and the material shows constant moduli for samples ≥40 kDa in film on water testing.^112^ The mechanical behavior of a conducting polymer is investigated by using various theoretical techniques like strain-induced elastic buckling instability. Hutchinson and co-workers developed a quantitative idea of the surface wrinkling patterns of stiff polymers. This technique is useful for the mechanical analysis of organic semiconductors, polymer films, carbon nanotubes, fibrous polymer materials, polyelectrolyte multilayer films, conjugated polymers, *etc.* The moduli of the system have been evaluated by placing the conjugated polymer coatings on soft substrates and related to bucking instability.vi
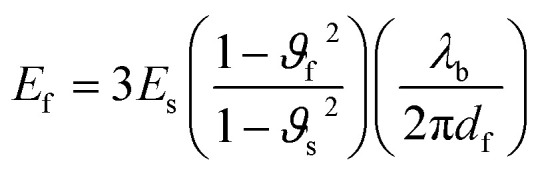
where *λ*_b_ is the buckling wavelength which is related to the polymer film thickness *d*_f_ and tensile moduli of both film and substrate *E*_s_ and *E*_f_ respectively. The Poisson ratios of both film and substrate are noted as *ϑ*_f_ and *ϑ*_s_. The Poisson ratios are assumed to be 0.5 and 0.35 for PDMS and conjugated polymer films, respectively. Seitz had developed a theoretical method for the determination of the mechanical behavior of polymers by considering molecular properties like length and rotational bonds present in the polymer, molecular weight, van der Waals, glass transition temperature, and length of rotational bonds. The model was modified for materials with glass transition temperatures above room temperature or below room temperature. The tensile modulus of the polymer is related to the bulk modulus and Poisson ratio.^113^

vii *E*_f_ = 3*B*(1 − 2*v*_f_)

The bulk modulus of the material has been given by the Lennard–Jones potential equation, which is attributed to the function of cohesive energy *E*_coh_ and molar volume *V* at room temperature.viii
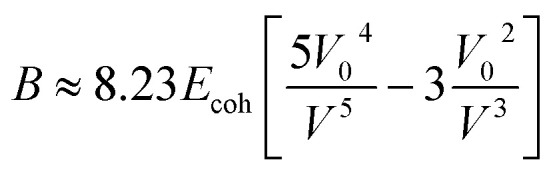
where *V*_0_ is the volume at the energy minimum. The cohesive energy from the molecular structure was evaluated using the Fedors method, and the molar volume has been calculated by using empirical correlation depending on the *T*_g_ value of the polymer. The value of molar volume depends upon the glass transition temperature: *i.e.*, *T*_g_ higher than room temperature, *T*_g_ close to room temperature, or *T*_g_ below room temperature.ix
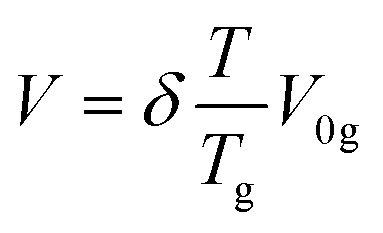
x
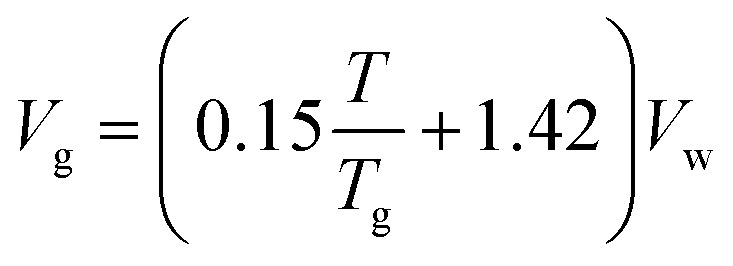
xi
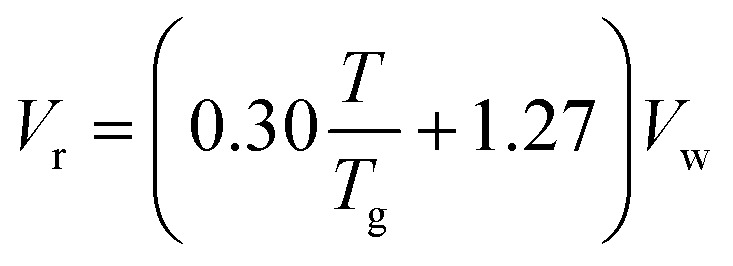


The Poisson ratio of the polymer is modelled with the molecular correctional area, which is given asxii
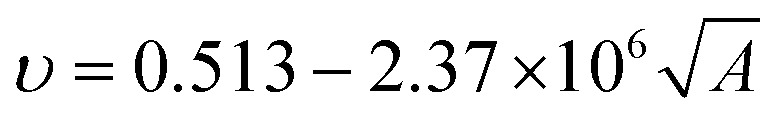


The molecular correctional area depends upon factors like van der Waals volume *V*_w_ and length of the monomer chain in its fully extended conformation.xiii
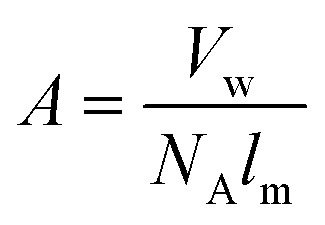
where *N*_A_ is Avogadro's number. Both *V*_w_ and *l*_m_ are estimated from the structure of the monomer.

### 5.2. Structure–property relationship

There are a lot of structural properties like side chain length, regioregularity, degree of polymerization, and presence of non-conjugated monomer units, which can affect the mechanical properties of conjugated polymers.^114^ The side chain attached to conjugated polymers results in novel polymers with unique physical and chemical properties. Attached aliphatic chains play an important role in the mechanical properties of conjugated polymers. The glass transition temperature of both engineering and conjugated polymers will decrease when attached to side groups. Gu *et al.* discovered that the mechanical properties of the conducting polymer polydiketopyrrolopyrrole will depend strongly on the length of the attached side chain. Here they observed that an increase in alkyl side group on the polymer backbone will reduce both the elastic moduli and tensile strength of the polymer.^115^ The hydrogen bonding present in the side chain also affects the mechanical and electronic properties of conducting polymers.^116^ Another factor that depends on the mechanical property is the bonding arrangement of the monomer unit present in the polymer. The increase in regioregularity of polythiophene and its derivatives will increase the tensile strength and decrease the polymer toughness and elasticity.^117,118^ The high degree of polymerization will enhance the chain entanglement in the polymer system and this entanglement will affect the physical properties. The entanglement will resist cavitation and crack formation. It also protects against the breakage of the polymer chain.^110,119^

## 6. Applications of conducting polymers

### 6.1. Supercapacitors

The world economy plays an important role in the utilization of fossil fuel sources, natural gas, and coal. The depletion of fossil fuels is causing a lot of social and environmental problems. Due to climate change, global warming, and health issues, the replacement of conventional energy sources is mandatory. Because of this, scientists have put their effort into developing eco-friendly, high-energy, renewable energy resources, including supercapacitors, fuel cells, and wind energy. Nowadays, supercapacitors are of great commercial interest because of the future markets for wearable devices, electric vehicles, *etc.* The main difference between conventional capacitor and supercapacitor devices is that they store 1000 times more energy than a dielectric capacitor. Also, they have a high-speed charge–discharge cycle, and they exhibit high energy and power density and also good cycle life.^120^

Supercapacitors are mainly of three types depending on their charge storage mechanism. In the case of an EDLC, it works by a non-faradic process, and the accumulation of charge happens in the interface between electrode and electrolyte. An electrochemical double layer of charge occurs and this double layer is responsible for capacitance. Carbon-based materials such as carbon, carbon nanotubes and graphene with high surface areas are mainly governed by this energy storage mechanism and are commonly used as electrode materials for EDLC. In the case of pseudocapacitors, they work by a faradic process, and charge storage happens by a redox reaction or intercalation process. Pseudocapacitors have increased power density and energy density compared to EDLCs.^121^ Conducting polymers^122^ and transition metal oxides such as Mn_3_O_4_ (ref. ^123^) or Co_3_O_4_ (ref. ^124^) have been extensively used as electrode materials for pseudocapacitors due to their redox activity. Hybrid supercapacitors are a combination of both EDLCs and pseudocapacitors. EDLCs show proper specific capacitance and good cycle stability and pseudocapacitors have good energy and power density. In hybrid capacitors, the advantages of both EDLC and pseudocapacitor are present.^75^

**Fig. 9:**
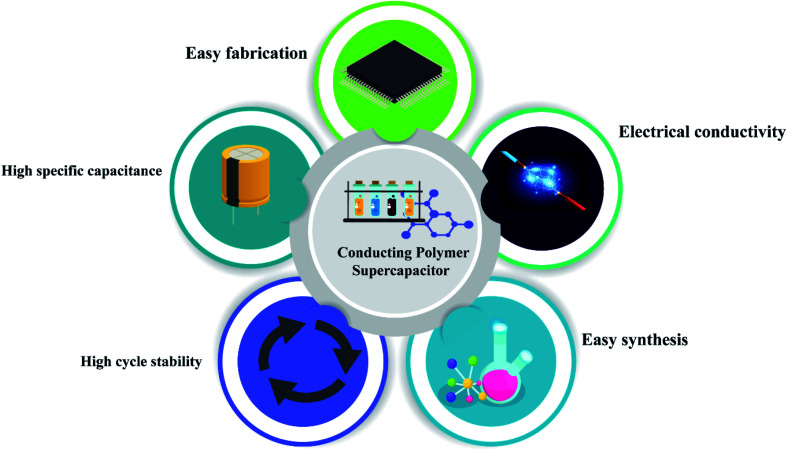
Schematic representation of basic properties of conducting polymer based supercapacitors.

**Fig. 10:**
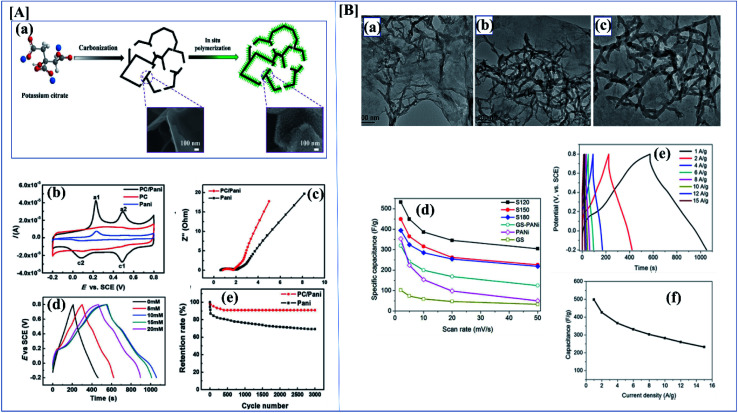
[A] (a) Synthesis route for PC/PANI composite. (b) CV curves of the electrodes of PC/PANI composites, PC, and PANI at 0.05 V s^−1^. (c) Nyquist plots of PC/PANI composite. (d) GCD curves of PC/PANI composites with different concentrations of aniline at 1.0 A g^−1^. (e) Cycle stability of composites at a constant current density of 5.0 A g^−1^ (reprinted with permission,^104^ copyright 2018 Wiley) [B] (a–c) TEM images of GO/PANI composites at different temperatures of 120, 150 and 180 °C. (d) Specific capacitance of GNS/PANI composites at different scan rates. (e) The GCD curves with different current densities of GO/PANI composites synthesized at 120 °C. (f) Specific capacitance of GO/PANI composites at different current densities (DOI: 10.1038/srep44562, Open source Nature).

Conducting polymers have many applications due to their electrical properties, high theoretical capacitance, good wave absorption,^125^ high redox activity, and excellent electrochemical behavior.^126^ Conducting polymers are available in different morphologies like sheets, particles, hydrogels, rod-like structure, *etc.*^127^ These different morphologies have different electrical, mechanical and optical properties (Fig. 9).^128^ The specific capacitance of a composite depends heavily on the material morphology. Zhang *et al.* investigated the synthesis and evaluated the performance of porous carbon nanosheets/polyaniline nanowires for high-performance supercapacitor applications. Generally, carbonaceous materials like graphene, activated carbon, or graphite are composited with polyaniline to overcome the cycle stability drawback of polyaniline. The main demerit of polyaniline carbonaceous composite materials is that they aggregate at the time of synthesis, which reduces the active sites and increases ion mobility, severely affecting the performance of the supercapacitors. To overcome this problem, researchers focused on 3D porous carbons.^129^ Here, the composite was synthesized by an *in situ* chemical oxidative method using ammonium persulfate. Aniline concentration also plays an important role here because aniline concentration increases the specific capacitance. Here polyaniline shows an antagonistic effect. The supercapacitance performance of the composite was studied using CV and GCD. From the electrochemical data, it was shown that the specific capacitance of PC/PANI is 512 F g^−1^, which is much higher than that of PC or polyaniline (250 and 201 F g^−1^) at a current density of 1.0 A g^−1^. From the asymmetric data, it was revealed that PC/PANI//PC has a proper capacitance of 77 F g^−1^ at a current density of 1.0 A g^−1^. The maximum energy density and power density are 17.1 W h kg^−1^ and 14 000 W kg^−1^ which are higher than previous reports (Fig. 10[A](a–e)).^130^

The mechanical stability of polyaniline is effectively utilized in the fabrication of flexible supercapacitor applications because of its easy synthesis, redox characteristics, conductivity, *etc.* Sharma *et al.* investigated the performance of a three-dimensional cellulose/graphite/polyaniline ternary composite. In this study, they followed a novel synthesis method to fabricate the supercapacitor. They pasted graphite sheets on top of cellulose paper by sandwiching them with PVDF. This flexible cellulose graphite sheet was further used for the electrodeposition of polyaniline. From the electrochemical data, we can see that it provides an excellent specific capacitance of 357 F g^−1^ in a three-electrode setup and a specific capacitance of 256 F g^−1^ in a two-electrode system at a current density of 100 mA cm^−2^. The high specific capacity is due to the higher number of active sites for a redox reaction in polyaniline nanowires. These supercapacitors show a high specific energy of 64.8 W h kg^−1^, an energy density of 6.48 W h L^−1^, and a cycle stability of 86% after 1000 cycles.^131^

Carbonaceous materials like CNTs, graphene and graphite have potential applications in different fields due to their structural, mechanical and electrical properties. SWCNT and MWCNT have been widely used in supercapacitor applications over the last decade.^132^ Simotwo *et al.* studied the synergetic effect of both CNT and polyaniline. They synthesized high-purity electrospun polyaniline and polyaniline/carbon nanotube fibers, and their supercapacitor performance was investigated. Electrospinning is a fiber production method using electric charge. Here poly(ethylene oxide) was used as a carrier polymer for electrospinning. The PANI : CNT : PEO weight ratio was kept fixed at 81 : 12 : 7. From the electrochemical data, CV shows that a polyaniline/CNT composite has good specific capacities of 324 and 281 F g^−1^ at 5 mV s^−1^. When the GCD current density was varied from 0.5 to 10 A g^−1^ PANI/CNT gave a specific capacitance of 380 F g^−1^ and polyaniline gave 308 F g^−1^ at a current density of 0.5 A g^−1^. From the CV graph, anodic and cathodic peaks in PANI/CNT undergo a slight shift to the left or right directions with increasing scan rate. This may be due to the internal resistance and also due to the phase transfer of polyaniline from the emeraldine salt to luecoemeraldine salt form in the presence of CNTs.^133^

As discussed before, morphology plays a vital role in the performance of a supercapacitor. It is possible to synthesize a variant morphology of polyaniline by controlling the hydrothermal conditions.^134^ Among the studied composites of polyaniline, polyaniline/graphene is of great industrial interest because of the synergetic π–π effect. Wang *et al.* evaluated the synthesis and SC performance of graphene polyaniline composites following two-step hydrothermal synthesis to obtain the composite. In this controlled synthesis, they tried different temperature ranges and the performance of each composite was evaluated. They first synthesized a GO/polyaniline intermediate suspension, and this was transferred into an autoclave and the reaction was carried out at various temperatures like 120, 150 and 180 °C. By analyzing Fig. 10[B](a–f), we can clearly understand that S120 gives excellent SC performance compared to the others. From the CV data, the GNS/PANI composite shows a proper capacitance of 532.3 F g^−1^ at a scan rate of 2 mV s^−1^ with good cyclic performance (capacitance retention is as high as ∼99.6%) after 1000 cycles.^135^

**Fig. 11:**
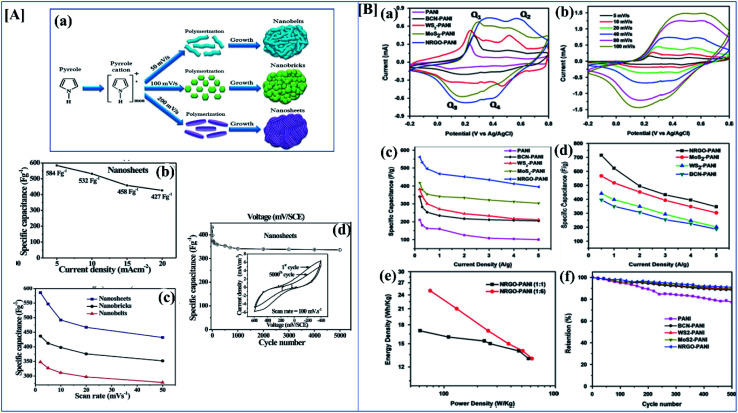
[A] (a) Schematic illustration of evolution of different morphologies of polypyrrole by electropolymerization. (b) Specific capacitance variation of polypyrrole nanosheets at different current density ranges. (c) Variation of the particular capacitance of nanobelts, nanobricks and nanosheets at different scan rates. (d) Change in specific capacitance of polypyrrole nanosheets with varying numbers of cycles (reprinted with permission,^105^ copyright 2012 Royal Society of Chemistry) [B] CV of (a) 1 : 1 nanocomposites of polyaniline with NRGO, MoS_2_, WS_2_ and BCN at 40 mV s^−1^ (b) CV of NRGO PANI composite at different scan rates. (c) Specific capacitance of 1 : 1 nanocomposites of PANI with NRGO, MoS_2_, WS_2_ and BCN at different current densities. (d) Specific capacitance of 1 : 6 PANI nanocomposites with NRGO, MoS_2_, WS_2_ and BCN at different current densities. (e) Ragone plots of 1 : 1 and 1 : 6 NRGO–PANI nanocomposites. (f) Cyclic stability of 1 : 1 nanocomposites of PANI with NRGO, MoS_2_, WS_2_ and BCN at a current density of 2 A g^−1^ (reprinted with permission,^127^ copyright 2014 Elsevier).

Nanomaterials with high surface area and good porosity have been suggested as good electrode materials for supercapacitors.^136,137^ Among conducting polymers, polypyrrole has good conductivity, good redox reversibility, high environmental friendliness, and mechanical stability. Due to these properties, polypyrrole is used in different applications like corrosion inhibition,^138^ sensors,^139^ supercapacitors, fuel cells,^140^*etc.* Nanostructures of polypyrrole can be easily synthesized by the direct electropolymerization of monomers on the substrate. Deepak P. Dubal *et al.* effectively investigated the structural and electrochemical properties of potentiodynamically synthesized polypyrrole. Here they synthesized different morphologies of polypyrrole by varying the electrochemical parameters (Fig. 11[A](a)). When the scan rate is increased, the morphology changes from nanobelts to atomically thin nanosheets. Analyzing the CV and GCD data from Fig. 11[A](b and c) clearly shows that the morphology plays an important role in the specific capacitance and stability after 5000 cycles. Here the nanobelt morphology has the lowest specific capacitance of 296 F g^−1^, the nanosheets possess a higher value of 584 mF g^−1^, and nanobricks have an intermediate value of 357 F g^−1^. Nanosheets possess a high discharge to charge efficiency of 91%.^61^ Ge *et al.* investigated the performance of rGO/PPy nanofilms. They were fabricated by vacuum filtration along with the electrochemical reduction of graphene oxide. This free-standing rGO/PPy film has both high mechanical and electrochemical properties. From electrochemical data, it shows a high areal capacitance of 216 mF cm^−2^ at 0.2 mA cm^−2^, and a high capacitance retention rate of 87% after 5000 cycles.^141^

Coaxial supercapacitors are very hard to fabricate because of their precise layer-by-layer arrangement of positive and negative electrodes. The main interest behind coaxial supercapacitors is they do not need any supporting materials and also that they have good ion transport between positive and negative electrodes. This is because of the short separation distance. Cheng *et al.* evaluated the performance of MnO_2_ nanosheets integrated with CNT/PEDOT:PSS coated CNT fibers. The PEDOT:PSS coating was done by continuous dipping of CNT fibers in PEDOT:PSS solution followed by annealing at 120 °C. The ternary CNT/PEDOT:PSS/MnO_2_ fiber electrode was achieved by the electrochemical growth of MnO_2_ on the CNT/PEDOT:PSS fiber. CNT/PEDOT:PSS provides both pseudocapacitance and binding action to combine both the outer MnO_2_ layer and inner CNT fiber. From electrochemical data, it is proven that CNT/PEDOT:PSS/MnO_2_ has an excellent specific capacitance value of 411.6 F g^−1^, better than that of CNT/MnO_2_. It provides a high capacitance retention rate of 91% over 10 000 cycles.^142^ Similarly, Yao and co-workers investigated the performance of a coaxial fiber shaped asymmetrical supercapacitor with a CNT core covered with vanadium nanowire arrays as the negative electrode material with an Na_2_SO_4_/PVA gel electrolyte. Here the positive electrode MnO_2_/PEDOT:PSS/CNT sheet was wrapped over the negative electrode. Initially MnO_2_ was deposited electrochemically over CNT fiber. From the electrochemical data, it delivers an excellent areal capacitance of 213.5 mF cm^−2^ with a corresponding energy density of 96.07 μW h cm^−2^ and excellent flexibility.^143^

Conducting polymers have utilized the advantages of metal oxides in many composites because of their high theoretical capacitance, high electrochemical activity and reversibility properties.^144^ Cobalt oxide has a low electronic property and the worst electrochemical cycle stability. To overcome these drawbacks, carbon matrices, like CNT, graphene, *etc.*, have been assembled. A hybrid with carbonaceous materials overcomes the above disadvantages of cobalt oxides. Raj *et al.* synthesized a Co_3_O_4_/polyindole composite *via* an electrodeposition method for supercapacitor applications. They chose polyindole because it gives higher thermal stability, higher redox activity, slower degradation and higher thermal stability than other conducting polymers like polyaniline or polypyrrole. The electrochemical properties of this hybrid system were studied by CV and GCD analysis. From the data, Co_3_O_4_/polyindole gives a very good specific capacitance of 1805 F g^−1^ while pristine Co_3_O_4_ has a specific capacitance of 1565 F g^−1^ at a current density of 2 Ag^−1^.^145^ This is much higher than previously reported for hybrids like CNT/Co_3_O_4_ (ref. 146) or Co_3_O_4_/graphene^147^ composites.

Transition metal dichalcogenides like MOS_2_, MoSe_2_, NbSe_2_, MoTe_2_ or WTe_2_ are promising materials for supercapacitor applications. They have a good electric double layer property, and they also have excellent mechanical and electrochemical stability.^148^ Pristine MoS_2_ itself gives very high specific capacitance and they also have different phases like 1T, 2H or 2R.^149^ I-Wen Peter Chen and his co-workers developed an ultrathin MoS_2_/PANI/CNT composite paper type flexible supercapacitor with an unusual volumetric energy density. Transition metal dichalcogenides have different phases according to which specific capacitance changes. They have synthesized the composite by a chemical *in situ* polymerization technique and different compositions have been tried. Among the synthesized composites, a composite with a high amount of polyaniline had a better specific capacitance than MoS_2_ and a composite with the right amount of MoS_2_ showed a high energy density of 0.013 W h cm^−3^ and an ultrahigh power density of 1.000 W cm^−3^. Most of the 2D materials have good electronic properties and good electrochemical activity.^150^ K Balakrishnan *et al.* synthesized different polyaniline-2D material composites, and composite performance was evaluated by electrochemical characterization techniques. We all know that 2D materials have novel structural and electrochemical activities, and that they show good specific capacitance in their pristine forms. Here they investigated polyaniline nanocomposites of few-layered MoS_2_, WS_2_, borocarbonitrides (B_*x*_C_*y*_N_*z*_), and nitrogen-doped rGO. Borocarbonitrides (B_*x*_C_*y*_N_*z*_) contain different proportions of boron, carbon and nitrogen, and they have a hexagonal BCN structure like graphene. The property of BCN changes with changes in the proportions of B, C, and N. They exhibit a composition-dependent electronic property. Depending on the composition, the band gap varies between 1.0 and 3.9 eV and a carbon-rich composition possesses a huge bandgap.^151^ In this work, they studied the supercapacitor behavior of polyaniline nanocomposites of MoS_2_, WS_2_, borocarbonitrides (B_*x*_C_*y*_N_*z*_), and nitrogen-doped rGO. Synthesis was through an *in situ* chemical polymerization technique, and electrochemical performance was evaluated in three-electrode systems with 2 M H_2_SO_4_ as the electrolyte. By understanding CV and GCD data, it is clear that the 1 : 1 composite has less specific capacitance and capacitance rises with increasing polyaniline content.^152^ Among the composites, the NRGO/polyaniline composite has a good cycle retention property. In the impedance data, it is clear that NRGO and MoS_2_ polyaniline composites have good capacitance properties in the lower frequency range. Pristine polyaniline possesses higher resistance than its composites (Fig. 11[B]).

MoS_2_ has a considerable interlayer distance. It possesses good chemical and electrochemical stability due to this. They are widely used in Li-ion batteries and supercapacitors. Interlayers are attracted by weak van der Waals forces. Intercalation is the easiest process to obtain monolayered MoS_2_. The monolayered MoS_2_ nanosheets are used as a template for growing conducting polymers. Wang *et al.* have developed a pizza-like MoS_2_/polypyrrole/polyaniline ternary nanostructure. This ternary hybrid system is synthesized by a two-step *in situ* chemical route. The main problem of conducting polymers is that they have very poor cycle stability. To overcome this problem, conducting polymers are used to make hybrid nanostructures with carbonaceous and other materials. In this study pristine MoS_2_ and MoS_2_/PPY show an ideal rectangular CV curve without characteristic redox peaks and a fast charge–discharge cycle. While polyaniline and binary hybrids of polyaniline show a CV curve with redox peaks. On analysis, it is clear that the MoS_2_/PPy/PANI ternary system has an enlarged area and it exhibits a good specific capacitance of 1273 F g^−1^ at 0.5 A g^−1^. Performance is maintained at 83% after 3000 charge–discharge cycles with high cycle retention.^153^

A summary of studies on the use of conjugated polymers in supercapacitor applications is given in Table 1.

**Table:** Summary of studies on the use of conjugated polymers in supercapacitor applications

Polymer	Electrolyte	Specific capacitance	Cycle stability	Energy and power density	Ref.
Polyaniline/graphene	EMITFSI/PVDF–HFP	87.8 mF cm^−2^ at 0.22 mA cm^−2^	100% after 10 000 cycles	12.2 μW h cm^−2^ and 226.4 μW cm^−2^	154
Polyaniline/graphene/Fe_2_O_3_ hydrogel	1 M H_2_SO_4_	1124 F g^−1^ at 0.25 A g^−1^	82% after 10 000 cycles	14.4 W h kg^−1^ and 58 W kg^−1^	155
MnO_2_/polyaniline/hollow mesoporous silica	PVA–KOH	248.5 F g^−1^ at 1 A g^−1^	>97.7% after 5000 cycles	88.4 W h kg^−1^ and 800 W kg^−1^	156
Polythiophene-graphite graphene oxide	1 M KOH	971 F g^−1^ at 1 A g^−1^	98% after 10 000 cycles	38.11 W h kg^−1^ and 7000 W kg^−1^	157
PEDOT:PSS/rGO	1 M H_2_SO_4_	249.5 F g^−1^ at 0.5 mA	75% after 2000 cycles	10.68 W h kg^−1^ and 81.25 W kg^−1^	158
NiCO-MOF/polypyrrole	2 M KOH	1109 F g^−1^ at 0.5 A g^−1^	79.1% after 10 000 cycles	41.2 W h kg^−1^ and 375 W kg^−1^	159
rGO/PEDOT/PANI	Solid electrolyte	388.5 F g^−1^ at 5 mV s^−1^	99% after 10 000	26.89 W h kg^−1^ and 800 W kg^−1^	160
SDBS doped polypyrrole/HC	6 M KOH	1086 F g^−1^ at 5 mV s^−1^	90% after 2500 cycles	218.05 W h kg^−1^ and 1075 W kg^−1^	161
Ti_3_C_2_/polypyrrole	2 M H_2_SO_4_	109.4 mF cm^−2^ at 1.05 mA cm^−2^	96% after 10 000 cycles	3.398 μW h cm^−2^ and 0.0845 mW cm^−2^	162
Carbonized iron/polyaniline/graphene Ni foam	1 M NaNo_3_	69.9 F g^−1^ at 1 A g^−1^	91% after 10 000 cycles	68 W h kg^−1^ and 718.2 W kg^−1^	163
CuS/C@polyaniline	3 M KCl	425.53 F g^−1^ at 1 A g^−1^	89.86% after 3000 cycles	—	164

### 6.2. Corrosion inhibition applications

Metals have potential applications in automobiles, construction, energy, household industries, packaging, and aerospace. Due to this, they have a perfect global market. When metals are exposed to moisture, or an acid or alkaline environment, they degrade very rapidly. The economic aspect of corrosion is unbelievable. In the USA and developing countries like Nigeria, 300 billion dollars and 10 billion dollars are respectively spent every year on research into and prevention of corrosion.^165^ Inspired by this, researchers have paid good attention and are focusing on corrosion–resistant coatings. Nowadays, a variety of corrosion inhibition techniques and coatings are available. In earlier years, chromate-based primers were quite extensively used because of their high corrosion–resistant property. Chromate coatings are used as both anodic and cathodic inhibitors. They have good adhesion with the metal surface and with topcoats and they are also economically feasible.^166,167^ The main demerit of chromates and chromium-containing coatings is that they are hazardous to both the environment and to human life (they are carcinogenic). For this reason, the Environmental Protection Agency (EPA) has limited the use of chromate conversion coatings.^168^ Nowadays, chromate conversion coatings have been replaced by organic coatings like epoxy,^169^ acrylic,^170^ polyvinyl butyral,^171^*etc.* Conducting polymers and conducting polymer pigmented paint coatings play a very potent role in corrosion inhibition. Conducting polymers have been receiving increasing attention, as possible components of corrosion–resistant coating systems, due to their ability to maintain the stable passivity of a metal by an anodization process coupled with O_2_ reduction on the surface of the film. They form a metal oxide interface layer between metal and coating. Also, in the case of phosphate pigments, specifically phenyl phosphonic acid, they can form a metal phosphate thin film on the metal surface, and this phosphate layer inhibits corrosion effectively.^172^

The mechanisms behind the corrosion inhibition property of polymers are given below.

1. Anodic protection mechanism: in this mechanism, polyaniline can generate a metal oxide layer above the metal surface and beneath the coating. These metal oxide layers act as a powerhouse for corrosion inhibition.^173^

2. When a metal comes into contact with conducting polymers, an electric field is generated. This electric field restricts the flow of electrons from the metal to oxidized species. Thus, corrosion inhibition occurs.^174^

3. Conducting polymers, especially polyaniline, have the property of controlled release of inhibitors upon reduction. A conducting polymer-based coating deposited on the metal surface will release the anion when reduction occurs. This property reduces corrosion.^175^

Among coatings, epoxy-based coatings are widely using in corrosion inhibition applications. Pigments are an important ingredient in these coatings, to achieve the desired application. As we discussed above, chromates are one of the main pigments used for corrosion inhibition. Due to their environmental and social problems, chromate conversion coatings are being replaced by other pigments. Graphene-incorporated coatings are also studied because of their hydrophobicity, sheet-like morphology, inertness, and because they act as a barrier to chemical aggressiveness. Zinc phosphates are also a very commonly used anticorrosion pigment because they are less toxic and have lower toxicity than chromates. Zhu and his co-workers investigated the synergetic effect of polypyrrole functionalized graphene and zinc phosphate pigmented epoxy coatings. From an electrochemical impedance spectroscopy study, it is clear that a coating containing a high amount of polypyrrole gives a good coating resistance *R*_ct_ value in 3.5% electrolyte. The *R*_ct_ values are 8.4 × 10^4^ Ω cm^2^, 7.7 × 10^4^ Ω cm^2^ and 2.5 × 10^4^ Ω cm^2^ with corresponding exposure times of 2, 24 and 240 h. From this we can observe that there is only an insignificant change in *R*_ct_. The addition of GO–PPy nanocomposites to the ZP coating accepts electrons and transitions from the oxidized state to the reduced state. The increased Fe^3+^ and Fe^2+^ ions are converted to a passive film of an oxide layer (Fe_3_O_4_ and Fe_2_O_3_).^176^

**Fig. 12:**
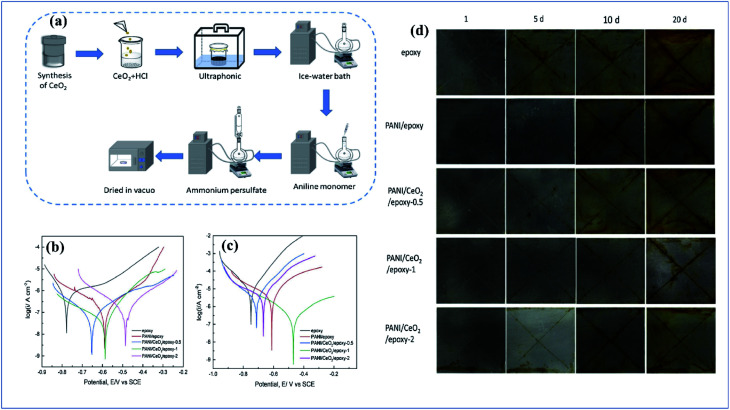
(a) Schematic representation of synthesis route for polyaniline/CeO_2_ nanoparticles. (b and c) Potentiodynamic polarization curve of control epoxy coating and conducting polymer composite pigmented coating after 1 and 15 days, respectively. (d) Salt spray test results after 20 days for epoxy coating, PANI/epoxy coating, PANI/CeO_2_/epoxy-0.5 coating, PANI/CeO_2_/epoxy-1 coating, and PANI/CeO_2_/epoxy-2 coating (reprinted with permission,^156^ copyright 2019 Elsevier).

Sathyanarayanan *et al.* developed polyaniline pigmented epoxy and vinyl acrylic organic coating, and evaluation of its performance was carried out on stainless steel. From their studies, they pointed out that the electrochemical impedance spectroscopy value initially decreases gradually; then the impedance value increases with an increase in exposure time. This phenomenon is due to the formation of pinholes on the surface of the metal surface. On increasing the exposure time, the coating adsorbed a metal-oxide interface layer in between the metal surface and beneath the coating.^177^ From their studies, they noticed that polyaniline pigmented organic coatings are very effective as corrosion inhibition coatings, and that this depends on the coating thickness and exposure time. They also observed that the corrosion resistance first decreases initially from 10^8^ to 10^6^ ohm cm^2^; then the coating resistance increases due to the passivity property of the polyaniline pigment.^178^ Lei *et al.* investigated the anticorrosion behaviour of polyaniline/CeO_2_ incorporated epoxy coating. Polyaniline/CeO_2_ was synthesized by a hydrothermal route followed by *in situ* polymerization. From analysis of the potentiodynamic polarization curve, the conducting polymer pigmented composite shows higher corrosion potential and lower corrosion current density than the control sample. The conducting polymer composite coating shows an improved anticorrosion tendency on a defective site after continuous salt spray analysis^179^ (Fig. 12[A]).

Electrochemically synthesized coating and polypyrrole pigmented organic coatings have also been explored. Armelin *et al.* utilized polypyrrole and polyaniline as anticorrosive additives in zinc epoxy coating, and their performance was evaluated *via* an immersion test and some standard ASTM tests. In this study, they made epoxy paint with and without the conducting polymer pigment and the performance of the coating was evaluated separately. From the research, they observed that it exhibits reversible redox properties like chromate coatings and this enhances its corrosion inhibition property. Zinc pigments generate zinc oxides which cover the metal layer and reduce the corrosion tendency, In this study, we can observe that by increasing the polypyrrole percentage beyond a certain limit, a better inhibition property is not exhibited. The percentage of polypyrrole must be optimum.^138^ Aguirre *et al.* studied the synthesis and performance evaluation of a poly(3,4-ethylendioxythiphene) PEDOT conducting polymer on a stainless alloy. They synthesized PDOT by cyclic voltammetry, giving 10 cycles in the potential range from −0.7 to 1.3 V at a scan rate of 0.05 V s^−1^. Here the corrosion inhibition property of the coated metal was studied electrochemically as well as through physical evaluation. Visual analysis and a peel test were undertaken in consecutive time intervals. Electrochemical analysis was done by OCP analysis from polarization curves. From the above characterizations, they concluded that PEDOT is an excellent promising material for the inhibition of corrosion.^180^

**Fig. 13:**
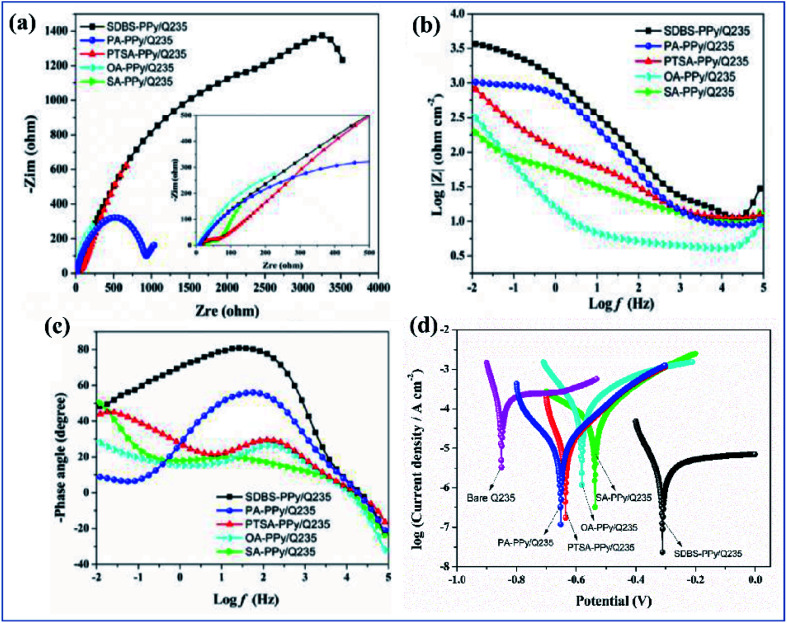
(a and b) Nyquist and (c) Bode plots of the prepared samples. (d) Tafel polarization curves of the bare metal and the coated polypyrrole films (DOI: 10.20964/2020.03.49, open source ESG).

Kalendova *et al.* discovered that epoxy paint contains polyaniline with acidic basic and neutral extracts like Zn_3_(PO_4_)_2_·2H_2_O, Ca_3_(BO_3_)_2_ and SrCrO_4_. From these studies, they observed that PANI + Zn_3_(PO_4_)_2_·2H_2_O has a good corrosion inhibition property in both NaCl and SO_2_.^181^ Electrochemically synthesized polypyrrole on Q235 steel with different dopants like oxalic acid, sodium dodecylbenzene sulfonate, toluene-*p*-sulfonic acid, sulfamic acid, or phytic acid was studied. The thickness of the coating was controlled to 40 μm. The anticorrosion behavior of all the above coatings was studied using dynamic potential polarization curves and electrochemical impedance spectroscopy. Fig. 13 clearly shows that polypyrrole coated metal is more suitable than bare metal. Among the above dopants, SDBS–PPy shows better anti-corrosion performance than other dopands.^182^

Many scientists have adequately studied the anticorrosion behavior of carbonaceous material. Carbon dots, carbon nanotubes, graphite, graphene and graphene oxide have been adequately investigated. It was observed that these materials possess a high rate of corrosion inhibition property. V. Martina *et al.* investigated the synergetic effect of a carbon nanotube polyaniline nanocomposite. Here studies were done through FTIR spectroscopic characterization before and after coating. Spectral analysis was utilized to understand the role of composite films in corrosion protection and to discriminate the best-performing CNTs.^183^ Electrochemically deposited polyaniline graphene nanofilms were synthesized along with the composite coatings and their cyclic voltammetry (CV) was investigated with a working electrode potential between −0.8 and 1.6 V at a scan rate of 0.05 V s^−1^ for 10 cycles. After electrochemical studies, they observed that the corrosion inhibition property of the polyaniline graphene composite is about 97%.^184^ Chemically synthesized polypyrrole–graphene oxide nanosheets blended with waterborne epoxy coating were studied. In GO–PPy nanosheets with zinc phosphate pigment (from the electrochemical impedance spectroscopy analysis) the coating process was found to have a very high rate of anticorrosion property compared to other coatings. This property is attributed to more complex arrays of non-agglomerated GO–PPy sheets and the passivation function of zinc phosphate in the coating.^176^ Montmorillonite polyaniline nanocomposite coatings also show outstanding corrosion inhibition behavior. The property of this composite using a series of potentiodynamic and impedance spectroscopy measurements in 3.5 wt% aqueous NaCl solution was investigated. From these studies, they discovered that the corrosion current (*i*_corr_) values decreased greatly.^185^

**Fig. 14:**
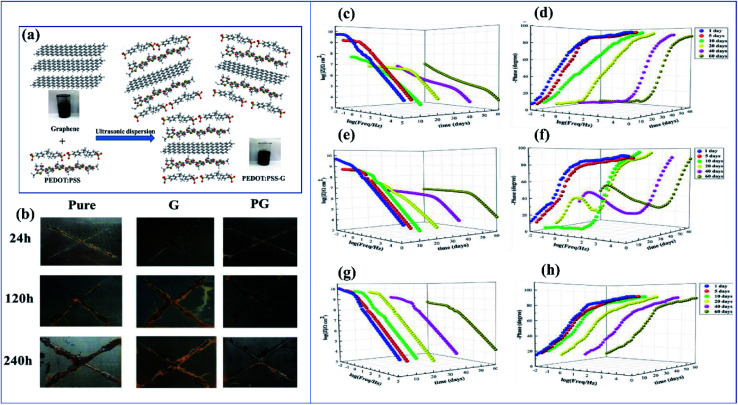
(a) Schematic illustration of the synthesis of PEDOT:PSS-G hybrids. (b) Optical photographs of pure-coating, G-coating, and PG-coating after salt spray test. The electrochemical impedance spectroscopy of (c, d) pure-coating, (e, f) G-coating and (g, h) PG-coating (DOI: 10.20964/2019.05.37, open source ESG).

Yue Su and co-workers developed a PEDOT:PSS-exfoliated graphene pigmented water-borne epoxy coating. PEDOT:PSS has an outstanding tendency to develop a metal oxide layer in between the topcoat and the metal surface, which protects the metal from degradation. As seen in Fig. 14(a), the π–π interaction between graphene and conducting polymer leads to exfoliation of graphene into monolayers. This will enhance the dispersion of graphene in the conjugate polymer matrix. The coated metal surface after a salt spray test reveals that the coating containing both conducting polymer and graphene protects the metal in a good way (Fig. 14(b)). Analysing the electrochemical data clearly shows that a graphene pigmented epoxy coated metal surface has a lower inhibition property than a pure epoxy coating and degradation occurs with increasing soaking time (Fig. 14(c–h)). Coating with both a conducting polymer and graphene gives a high impedance value. The coating develops a metal oxide layer after soaking.^186^

Corrosion is primarily due to atmospheric exposure and the electrochemical activity of metals. Corrosion inhibition is mainly attributed to the incorporation of an anticorrosive pigment and the placing of a physical barrier to prevent moisture contact. Hydrophobic coatings are preferred for their water-repellent properties. PANI/TiO_2_ coating possesses good thermal conductivity, good hydrophobicity, and good anti-corrosion activity. Thermal conductivity plays a vital role in the durability of a coating because thermal conductivity increases heat dissipation. It prevents the delamination of a coating resulting from heat expansion. Analyzing EIS data, it is clear that the PANI/TiO_2_ coating exhibits a greater area in the Nyquist plot. This means that the composite provides better corrosion inhibition. The hydrophobicity of a coating is evaluated by contact angle measurements. If the contact angle is below 90°, the coating must be hydrophilic and if contact angle is above 90°, the coating shows hydrophobicity. Here PANI/TiO_2_ has a contact angle of 150°, so it is clear that the coating is super-hydrophobic in nature.^187^

Before painting metal substrates, a chromate treatment or wash primer coat will be applied. Due to their toxicity and environmental issues, chromate conversion coatings have almost been replaced by wash primers. Generally, wash primers are made from polyvinyl butyral resins. The wash primer itself acts as a corrosion inhibition coating. To obtain a higher amount of anti-corrosion performance, polyaniline incorporated wash primers are being studied. In this work polyvinyl butyral containing polyaniline and chromate pigments are studied separately. By analyzing the electrochemical impedance data for 1–10 days in 3% NaCl electrolyte, it is clear that a polyaniline pigmented wash primer has a coating resistance value of 4 × 10^3^ to 2.3 × 10^3^ which is almost the same as that of a chromate pigmented primer (1.4–5.19 × 10^4^).^188^

A summary of studies on the use of conjugated polymers in corrosion inhibition is given in Table 2.

**Table:** Summary of studies on the use of conjugated polymers in corrosion inhibition applications

Substrate	Organic solvent	Anticorrosion pigment	Reference
Steel	Water-borne epoxy	Polyaniline/poly(methyl hydrosiloxane)	189
Carbon steel	Epoxy	Polyaniline/F-silicon nitride	190
Glass	Epoxy	Fluro substituted polyaniline	191
Carbon steel	Epoxy	Polyaniline–CNT	192
Stainless steel	Solvent free	Polypyrrole/functionalized carbon powders	193
Carbon steel	Epoxy	Polypyrrole functionalized zinc oxide and zinc phosphate	176
Porous stainless steel	Solvent free	Polypyrrole	194
Stainless steel	Sulfonated melamine formaldehyde	Polypyrrole	195
Aluminium	Solvent free	Polypyrrole	196
Magnesium alloy	Epoxy	Polyaniline@MIL-101	197
Carbon steel	Epoxy	2-Hydroxyphosphonocarboxylic acid doped polyaniline	198
Aluminium alloy	Solvent free	Ag/polyaniline	199
Magnesium alloy	Solvent free	Polypyrrole/V_2_O_5_	200

### 6.3. Photocatalytic applications

**Fig. 15:**
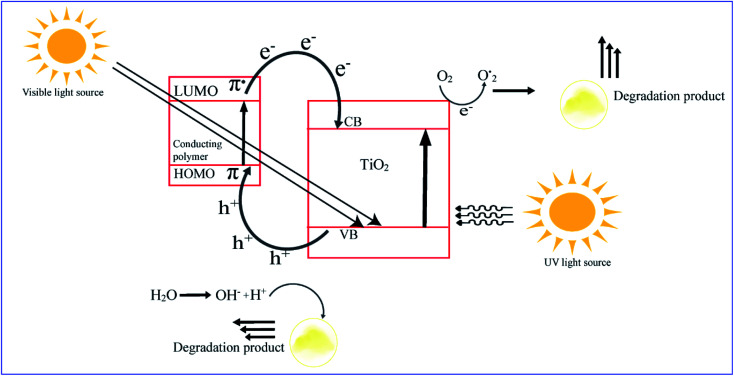
Photocatalytic mechanism in conducting polymer composites.

Among photocatalysts, TiO_2_ is preferred because of its novel bandgap, easy synthesis, cheapness, high photo-activity, resistance to photocorrosion and high oxidizing power. Generally, TiO_2_ exhibits three different phases: anatase, rutile, and brookite. Among the three phases, anatase has good photochemical behavior due to its high bandgap of 3.2 eV. Titanium dioxide can be easily prepared hydrothermally or by a sol–gel approach. Polyaniline with organic semiconductors is a great research prospect because it has good electron–hole charge separation, a good absorption coefficient of visible spectra, a high mobility of charge carriers, and low-cost and easy synthesis. The process behind dye degradation includes incidence of light on the photocatalyst, photo-induced holes, the accumulation of electrons on the semiconductor surface, initiation of a redox reaction, and degradation of the reaction product. Reddy and his coworkers synthesized a TiO_2_/polyaniline hybrid system by an *in situ* polymerization technique. They studied the photocatalytic activity of the composite for the degradation of rhodamine, acetic acid, and methylene blue. Under continuous 3 h UV irradiation, RhB was degraded by 80%, and methylene blue and phenol were degraded by 67% and 51%, respectively, in 200 min of irradiation time. The photocatalyst still gives good activity after 3 cycles of irradiation. It is clear that the catalyst has a reusable property. In the photocatalytic mechanism of TiO_2_/PANI studied by ESR spin trapping experiments, under UV irradiation, the main oxidative species are superoxide anion radicals, O_2_˙^−^, hydroxyl radicals, (˙OH) and holes (h^+^). When polyaniline is exposed to UV irradiation, a π–π* transition happens. This means that photogenerated electrons become excited to the π* orbital of polyaniline. Which has the same energy as the conduction band of titania, resulting in chemical bonding. This helps in the transfer of a photogenerated electron from polyaniline to TiO_2_. These absorbed electrons migrate to the surface of the photocatalyst and react with water and oxygen to produce hydroxyl and superoxide anion radicals, which cause oxidation of the contaminants. Similarly, photogenerated holes migrate from the valance band of TiO_2_ to the high energy state of polyaniline, because the VB of TiO_2_ and the CB of polyaniline have the same energy level. And these photogenerated holes migrate to the surface of the photocatalyst, and oxidation of contaminants occurs^201–205^ (Fig. 15).

Heavy metals like Zn, Pb, Hg, Cd, Cr, Ni, Co, Fe, *etc.* are harmful to the environment. These heavy metals are expelled as industrial waste and they dangerously affect the aquatic ecosystem. The US Environmental Protection Agency, Agency for Toxic Substances and Disease Registry listed chromium as a potentially toxic element.^206^ Chromium has a high amount of toxicity, which affects human life severely causing eczema, and lung, nasal, and sinus cancers, *etc.* Chromium exists in different valance states from −2 to +6. Among these, chromium[iii] and chromium[vi] are too dangerous due to their environmental stability.^207^ There are a lot of biological techniques used for the removal of chromium traces like bioremediation using bacteria,^208^ fungi^209^ and algae, and phytoremediation. By comparing these studies, physiochemical treatments like photocatalysis, adsorption,^210^ membrane separation^211^ and ion exchange^212^ can be used as effective methods for the removal of traces of chromium. A mesoporous titanium polyaniline nanocomposite can remove chromium ions. The mesoporous TiO_2_ can react with atoms, molecules and nanoparticles not only on the surface but also throughout the bulk. Deng *et al.* tried to point out the photocatalytic activity of the MT/PANI hybrid system for the successive removal of chromium ions. Here they synthesized a hybrid with different concentrations of polyaniline. The hybrid system is twice as active as pristine MT, and 3% MT/PANI adsorbed 100% of chromium[vi] ions after 10 cycles. It is evident that the composite has reusability. But MT and other concentrations of polyaniline had reduced activity after 10 cycles of reaction. This ideal efficiency is due to the adsorption behavior of the protonated amino group present in the polyaniline.^202^ The mechanism behind the photocatalytic activity of the polyaniline graphene composite has been beautifully described. When visible light is irradiated on the composite, the photogenerated electrons in the conduction band of polyaniline migrate towards the Fermi level of graphene. This is because the energy of both the CB of polyaniline and the Fermi level of graphene is almost the same. Then the migrated electrons on the surface of graphene react with the dissolved oxygen to form superoxide radical ions. These react with water to form H_2_O_2_. H_2_O_2_ reacts with the photogenerated electrons on the surface of graphene and photogenerated holes in the CB of polyaniline. The generated ˙OH is responsible for dye degradation. When the dye is exposed to UV irradiation, it is converted to dye*. In the case of MB, the photogenerated electron in the LUMO migrates to the VB of polyaniline. Then the photogenerated holes in the dye react in the presence of water to form ˙OH, which degrades the MG dye. In the case of RhB* and CR*, electrons can be injected into the CB of polyaniline *via* electron transfer and these electrons, in turn, flow downhill to RGO, and are scavenged by the O_2_ on the surface of the catalyst to form superoxide radical anions.^204^

Mechanism

18 PANI → (h_VB_^+^ + e_CB_^−^)PAN

19 (e_CB_^−^)PANI → RGO(FL)(e^−^)

20 (e_FL_^−^)RGO + O_2_ → ˙O_2_^−^

21 H_2_O + ˙O_2_^−^ → ˙OOH + OH^−^

22 ˙OOH + H_2_O → ˙OH + H_2_O_2_

23 H_2_O_2_ + (h_FL_^−^) → ˙OH + H^+^

24 H_2_O_2_ + (h_VB_^+^) → ˙OOH + H^+^

25 H_2_O_2_ + ˙OOH → ˙OH + H_2_O_2_ + O_2_

26 dye → dye*(e_LUMO_^−^)

For MG

27a dye* + (h_VB_^+^)PANI → dye* + PANI_VB_

27b H_2_O + (h_HOMO_^+^)dye* → ˙OH

For RhB and CR

27c dye* → dye^+^ + PANI/RGO(e_FL_^−^)

27d (h_VB_^+^)PANI + dye → dye^+^ + PANI

˙OH + ˙O_2_− + dye^+^ → colorless degraded product

**Fig. 16:**
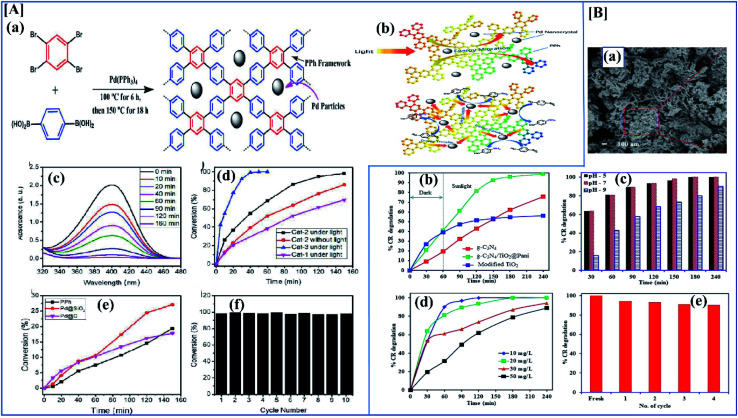
[A] (a) Synthetic route. (b) The process of light-harvesting and energy migration in PPh framework and the energy transfer to Pd sites for catalysis. (c) UV-vis absorption of the reaction mixture with 400 mg L^−1^ of Cat-2 in 2 mM of *p*-NPh under visible light. (d, e) Conversions of *p*-NPh to *p*-APh by different photocatalysts. (f) The catalytic activity of recycling with reaction time of 150 min (reprinted with permission,^192^ copyright 2013 Royal society of chemistry) [B] (a) SEM images of g-C_3_N_4_/TiO_2_@PANI nanocomposite photocatalytic degradation of CR. (b) Comparative degradation study. (c) Effect of solution pH on CR degradation onto g-C_3_N_4_/TiO_2_@PANI nanocomposite. (d) Effect of initial CR concentration on photocatalysis by g-C_3_N_4_/TiO_2_@PANI nanocomposite. (e) Reusability study g-C_3_N_4_/TiO_2_@PANI nanocomposite (DOI: 10.1038/s41598-019-48516-3, open source Nature).

Polyphenylene is widely used as a photocatalyst due to its high chemical and thermal stability. Gu and his team reported catalytic behavior in a Pd/polyphenylene composite synthesized by a palladium catalyzed Suzuki reaction. They observed that polyphenylene has a high light harvesting property along with energy transfer through its conjugated backbone. The reduced size effect of palladium causes a better catalytic reaction. From the UV-vis absorption data it is clear that the catalyst has better light absorption and the composite has higher conversion efficiency in the transition of *p*-NPh to *p*-APh. The Pd/polyphenylene catalyst shows better reusability after a series of reaction cycles. The light energy harvesting of polyphenylene causes the migration of electrons towards the palladium surface, resulting in a high electron density over the palladium surface. It also causes better hydrogen activation and thereby better photocatalytic activity (Fig. 16[A]).^213^

**Fig. 17:**
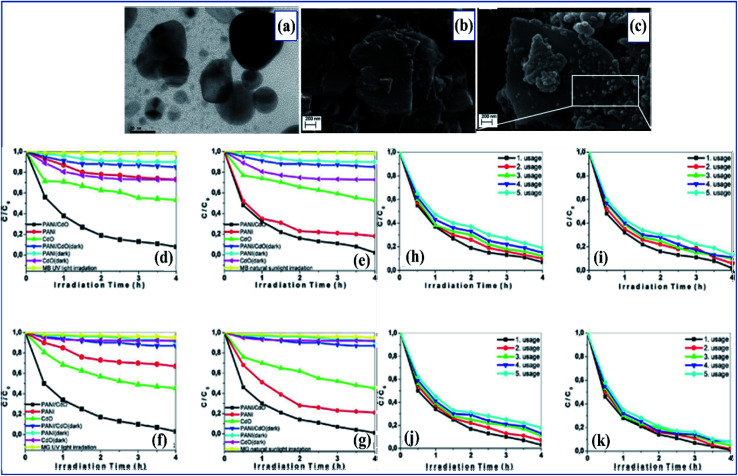
(a) TEM image of bare CdO nanoparticles. (b & c) SEM image of PANI homo polymer, and PANI/CdO nanocomposite. (d & f) Degradation of MB and MG under UV light irradiation by using PANI homo polymer and PANI/CdO photocatalyst. (e & g) Degradation of MB and MG under natural light irradiation by using PANI homo polymer and PANI/CdO photocatalyst. Effect of a number of runs on the degradation of dyes in the presence of PANI/CdO nanocomposite: (h) UV light irradiation of MB, (i) natural sunlight irradiation of MB, (j) UV light irradiation of MG, and (k) natural sunlight irradiation of MG. Catalyst concentration, 0.4 mg mL^−1^; initial concentration of dyes, 1.5 × 10^−5^ M (reprinted with permission,^183^ copyright 2013 American Chemical Society).

A chemically prepared polyaniline/CdO nanocomposite has an excellent dye degradation ability under both UV and sunlight irradiation. Electron densities and bond energy on the surface of the polyaniline while making a composite with CdO enhance the dye decoloration up to 7 times more than pristine polyaniline. The mechanism behind the degradation of methylene blue and malachite green has been emphasized. Both these dyes are extensively used industrially and biologically as well as chemically. These highly toxic dyes have been degraded using semiconducting materials like ZnO and TiO_2_. CdO has a high direct bandgap of 2.3 eV and an indirect bandgap of 1.8 eV. Therefore, it is a great research prospect in the field of optoelectronics, such as solar cells, phototransistors, photodiodes, transparent electrodes, catalysts, and gas sensors. In this study they examined the decolorization of dye by using polyaniline, CdO and polyaniline/CdO nanoparticles in the presence of natural sunlight as well as under UV irradiation. From the data it is clear that CdO concentration should be an optimum to obtain high efficiency of dye degradation. The kinetics of degradation are well described by the apparent first-order rate equation ln(*C*_0_/*C*_*t*_) = *κ*. A polyaniline/CdO composite has a higher *κ* value. The catalyst degrades 99%, and 98% of MB and MG dyes, respectively, in the first cycle of reaction. The catalyst has high reusability because it shows an efficiency of 82% and 97% after the fifth cycle^205^ (Fig. 17).

The photocatalytic behavior of the g-C_3_N_4_/TiO_2_@PANI composite was investigated. The composite has better photocatalytic activity against Congo red toxic dye due to the better electron–hole pair separation and reduced band gap of the composite. This was observed from UV-Vis analysis, PL and band gap analysis (Fig. 16[B]). The composite shows almost 100% photocatalytic activity towards Congo red molecules at some particular concentrations. The composite does not show active photocatalytic behavior beyond a particular concertation of Congo red due to the lack of optical density and a deficiency of active radicals to activate photocatalysis.^214^ The photocatalytic activity of polypyrrole with TiO_2_ nanoparticles for the degradation of organic dyes has also been studied. The energy level between the VB and the CB is denoted as the bandgap *E*_g_. Materials get excited only when the energy of an incident photon is equal to or higher than the *E*_g_. The electrons absorb energy from the photons, and they are excited to the CB from the VB. But in the case of TiO_2_, it has a wide energy gap. Due to this, the excitation in sunlight is limited and excitation shows best under UV irradiation. When making a composite with conjugative polymers, the bandgap is reduced compared to pristine TiO_2_. This is why a polymer TiO_2_ composite absorbs more photons, and the degradation of dyes is enhanced. The photocatalytic mechanism is almost the same here as in the case of polyaniline. Under sunlight irradiation the electrons in Ppy/TiO_2_ are excited from the highest energy state of polypyrrole into the lowest energy level. Then they will migrate into the CB of TiO_2_. The holes in the polypyrrole are left as such in the HOMO. The electrons in the VB of TiO_2_ move to the HOMO of polypyrrole and holes are left in the VB of TiO_2_, so the recombination of electrons–holes occurs. This means a huge amount of electron–hole generation takes place in the surface of the TiO_2_. These photogenerated holes and electron react in the presence of oxygen and water to form photoinduced anions like O^2−^ and OH^−^. These two photoanions are responsible for dye degradation. Here in this study Wang and co-workers investigated the performance of the polypyrrole/TiO_2_ in a (1 : 100) molar ratio. The first-order rate equation was used to determine the rate of degradation. From the data, it is clear that polypyrrole/TiO_2_ (1 : 100) has a higher *κ* value.^203^

The electronic property of a conducting polymer is easily tunable when a composite is made with metallic semiconductors. Pristine conducting polymers have problems with the high rate of electron–hole pair recombination, so it is mandatory to modulate this property to enhance photocatalytic activity. g-C_3_N_4_ has a moderate band gap energy, so it is easily excited under sunlight. Polyaniline has an outstanding electron-donating tendency and is an excellent hole acceptor. A hybrid of polyaniline and g-C_3_N_4_ has a decreased rate of electron–hole recombination and a unique production of photocatalysts so that the above composite is extensively using in the photocatalytic degradation of organic dyes. The above composite has been investigated for the degradation of methylene blue. 92.8% degradation and a high cycle life of the catalyst have been observed. Alenizi *et al.* have synthesized a novel ternary g-C_3_N_4_/TiO_2_@polyaniline composite and investigated the degradation of Congo red dye under sunlight. The deterioration of Congo red was studied previously by using WO_3_–TiO_2_/activated carbon, alginate/Fe_2_O_3_/CdS and TiO_2_-SWNT-P-21 composites, but the efficiency of degradation was not more than 85%. But here in this study, Congo red dye was decolorized by 100% in 3 h of reaction under sunlight. The composite has a higher rate of reusability after the fourth cycle with an efficiency of 90.1%. The mechanism behind this composite was the same as before. When it was exposed to sunlight, electrons were separated from the composite, migrated from the CB of TiO_2_ to the valence band of g-C_3_N_4_, and recombination with holes occurred. Generated holes migrate towards the surface of the polyaniline and the electrons left in the conduction band of g-C_3_N_4_ react in the presence of oxygen, H_2_O and OH^−^ to form photoanions. These photo-induced products drive the photocatalytic reaction. The exciting factor behind this composite is its higher efficiency compared to its pristine form or a binary composite of the above materials.^214^

A summary of studies on the use of conjugated polymers in photocatalytic applications is given in Table 3.

**Table:** Summary of studies on the use of conjugated polymers in photocatalytic applications

Polymer	Light source	Pollutant	Efficiency	Ref.
Polyaniline/TiO_2_	UV irradiation	Bisphenol-A	99.7% in 80 min	215
PPy–TiO_2_	Xe lamp (vis)	Methyl orange	100% in 60 min	216
BiVO_4_/Ag_3_PO_4_/PANI	Visible light illumination	CIP	85.92% in 60 min	217
PPy–ZnIn_2_S_4_	Iodine gallium lamp		100% in 60 min	218
CuO/TiO_2_/PANI	Visible light illumination	Chlorpyrifos	95% in 90 min	219
BiOBr–Ag–PPy	Halogen lamp (vis)	Malachite green	97% in 120 min	220
PANI/PbS QD	Visible light illumination	Rhodamine 6G	87% in 50 min	221
Polythiophene/Bi_4_O_5_I_2_	Visible light illumination	Bisphenol A	98.6% in 20 min	222
Polypyrrole/halloysite nanotubes/Fe_3_O_4_/Ag/Co	Visible light illumination	Methylene blue	91% in 120 min	223
Polythiophene/ZnO	Mercury lamp	(MB) dye and gemifloxacin mesylate (GFM) antibiotic	95% of MB dye 80% GFM in 180 min	62
ZnFe_2_O_4/_Ag/PEDOT	Sunlight irradiation	Tetracycline	71.77%	224
Polythiophene/MnO_2_	Visible light irradiation	MG dye and mixture of MG dye and trichlorophenol	98% for MG dye and 97% in 80 min	225

### 6.4. Biomedical and antimicrobial application

**Fig. 18:**
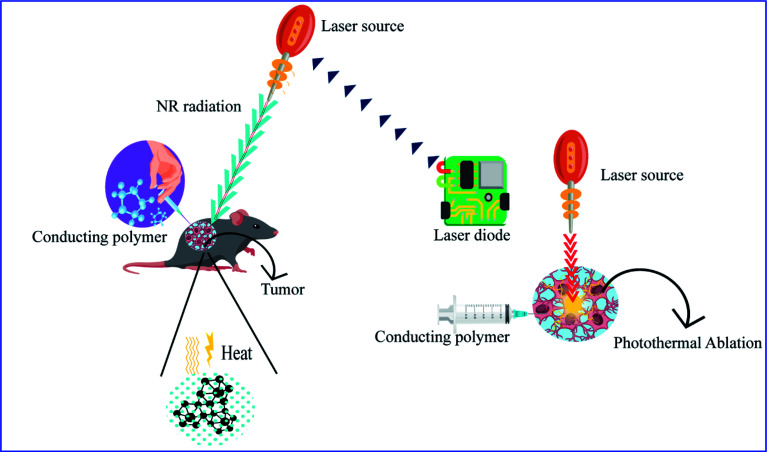
Schematic representation of photothermal treatment using a conducting polymer.

Near-infrared (NIR) photothermal treatment (PTT) is a promising approach for ablating cancer cells in the human body. An NIR laser is used to illuminate the targeted area using fiber optics. Ablation of cancer cells is done by the use of a photothermal material with minimal invasiveness compared to other treatment methods like chemotherapy or radiotherapy. One of the main advantages of PTT over other treatments is its targeted action. Due to this property, this treatment protocol minimizes the loss of tissues near the active site. Materials with nanoscale dimension, good NIR absorption, a high amount of photostability, and less photocorrosion and low cytotoxicity show great prospects as photothermal materials for PTT. The metal nanoparticles tend to show surface plasmon resonance when illuminated with IR radiation and, after excellent illumination, the photostability of most metal nanoparticles is lost. Conducting polymers like polyaniline, polypyrrole, PDOT (Fig. 18) *etc.* have outstanding capability for absorbing IR, and they have notably less cytotoxicity. That is why these polymers are widely used for both *in vivo* and *in vitro* cell studies. Mei Chen *et al.* studied the possibility of polypyrrole as a photothermal material for the control of the growth of the 4TI tumor model. In this study, they investigated it experimentally by *in vivo* and *in vitro* analysis, and they observed that polypyrrole has a photothermal efficiency of 44.7% and has an excellent cancer cell ablation property under NIR irradiation of 1 W cm^−2^. The tumor size was measured in consecutive intervals, and it was observed that all irradiated tumors disappeared, and no tumor growth was noted after a course of 60 days. The NIR photothermal treatment of F127 modified polyaniline was also studied. The composite was synthesized by a two-step hydrothermal method followed by colloidal dispersion of the composite by sonication.^226^

**Fig. 19:**
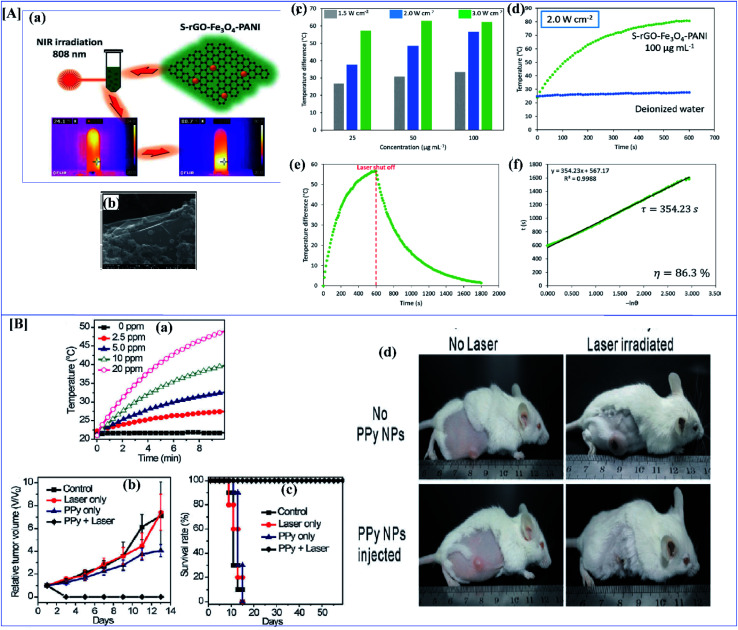
[A] (a) Schematic illustration of effect of NIR radiation on S-rGO–Fe_3_O_4_–PANI composite. (b) SEM image of S-rGO–Fe_3_O_4_–PANI. (c) Obtained maximum temperature difference values for S-rGO–Fe_3_O_4_–PANI with different concentrations at various laser power densities. (d) Heating curves for S-rGO–Fe_3_O_4_–PANI and deionized water at 2.0 W cm^−2^ laser power density. (e) Heating and cooling curves for S-rGO–Fe_3_O_4_–PANI at 2.0 W cm^−2^ laser power density. (f) −ln *θ*−*t* for the cooling period of S-rGO–Fe_3_O_4_–PANI after irradiation (reprinted with permission,^208^ copyright 2019 Elsevier) [B] (a) photothermal effect of pure water and PPy NPs with different concentrations upon irradiation by a 1 W cm^−2^ 808 nm laser. *In vivo* photothermal therapy study using intravenously injected PPy NPs. (b) Tumour growth rates of groups after different treatments. (c) Survival curves of mice bearing 4T1 tumour after various treatments. (d) Representative photos of tumours on mice after various treatments (reprinted with permission,^206^ copyright 2012 Royal Society of Chemistry).

Synthesized polyaniline composites have a potential future in photothermal treatment due to their high amount of strong NIR absorption, optimum size, good water dispersion, and high photothermal efficiency of 48.5%. The critical feature of zero toxicity is useful in in vivo investigations.^227^ Carbonaceous materials like graphene, graphene oxide, and reduced graphene oxide have a potential role in PTT because of their suitable NIR absorption, high surface area, and efficient thermal conversion, but their application has been limited because of their low dispersity. Recently magnetic iron oxides have also been used in various biomedical applications like targeted drug delivery, bioimaging, and even for cancer treatments. Soysal *et al.* investigated the synergetic behavior of an S-rGO–Fe_3_O_4_–polyaniline composite, and they observed that the composite has good photothermal as well as targeted drug delivery and bioimaging behavior. The composite irradiated at 808 nm has an excellent photoconversion efficiency of 86.3% and the composite with the least aqueous concentration has low cytotoxicity. It also shows an excellent bioimaging property (Fig. 19[A]).^228^

Polypyrrole has high photothermal activity towards cancer cells. Chen and his coworkers investigated the photothermal efficiency of polypyrrole towards 4T1 cancer cells without any surface modification. The biocompatibility of polypyrrole paves a way for both *in vivo* and *in vitro* analysis. From investigation they observed that polypyrrole has 44.7% photothermal conversion efficiency and it has a high ablation efficiency due to its high NIR absorption^226^ (Fig. 19[B]).

**Fig. 20:**
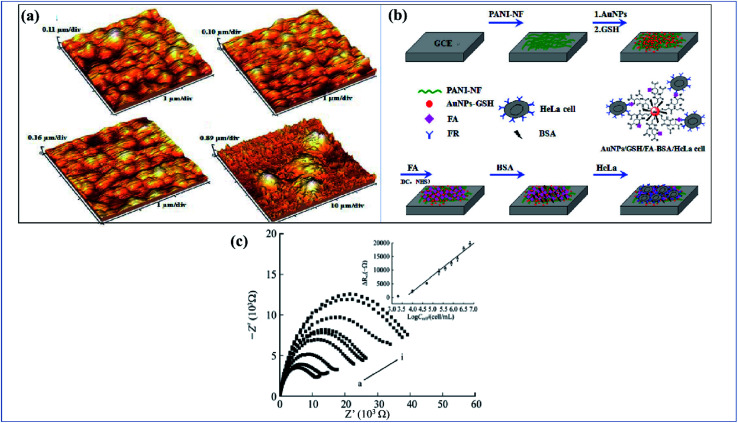
(a) AFM images of PANI-NF/AuNP surface, PANI-NF/AuNP/GSH surface, PANI-NF/AuNP/GSH/FA surface, and HeLa cells adhered on sensor surface. (b) Preparation of PANI-NF/AuNP/GSH/FA-BSA film and an impendence sensor with HeLa cells. (c) Nyquist diagrams of GCE/PANI-NF/AuNP/GSH/FA-BSA obtained with different HeLa cell concentrations (from a to i). Inset: a plot of EIS *versus* the logarithm of HeLa cell number (reprinted with permission,^209^ copyright 2010 Elsevier).

Cytosensors are used to analyze and evaluate cells by using electrochemical parameters like current, impedance, and capacitance. Cell activity concentration and other mechanisms like the proliferation of cells are adequately assessed by electrochemical sensors. Generally, zero-cytotoxic metal and magnetic and photochromic nanoparticles are applied for the investigation of cell studies beyond conducting polymers. Due to their high conductivity, electrochemical stability and response, conducting polymers have excellent potential as bio-electrochemical sensors. Cancerous HeLa cells impregnated with GCE/PANI-NF/AuNP/GSH/FA–BSA systems were investigated. Here the sensing action for HeLa cells by the composite is discovered in the changing impedance data. The HeLa impregnated composite restricts the flow of current from the composite to the electrolyte; hence an overall change in the EIS data is noted. From the impedance data, the potential of composites with and without HeLa cells was studied by the difference in the *R*_ct_ value. The impregnated composite has the lowest value of *R*_ct_ and has good composite resistance while the HeLa impregnated composite has a high *R*_ct_ value due to hindrance of the current flow to the electrolyte (Fig. 20).^229^

Conducting polymer fabrics show antimicrobial activity, so they have potential applications in biomedical as well as in different smart materials. They can increase the H_2_O_2_ level, which encourages the production of hydroxyl radicals which leads to antimicrobial action. Phytic acid doped polyaniline coated fabric has been studied in which phytic acid provides better crosslinking between polyaniline and the substrate. The antimicrobial action depends on the electrostatic attraction between the polymer backbone and the microbes. The conductivity changes with different values of pH: pH ≥3 gives better conductivity. The antibacterial activity of polyaniline coated cotton and polyester fabric against Gram-positive *Staphylococcus aureus* (*S. aureus*) and Gram-negative *Escherichia coli* (*E. coli*) and *Klebsiella pneumonia* (*K. pneumonia*) incubated for 18 hours at 37 °C was investigated in Mueller–Hinton agar (MHA). The investigation reveals that polyaniline coated polyester shows a better inhibition zone of 10.3 ± 0.04714, and cotton fabric shows no inhibition zone in the case of *Escherichia coli* (*E. coli*) or *Klebsiella pneumonia* (*K. pneumonia*).^230^

Conducting polymers are extensively used as an antimicrobial and antifungal agents in various fields like the food industry, the medical field, coating industry, *etc.* Polyaniline and other conducting polymers have an antimicrobial property against *Escherichia coli*, *Staphylococcus aureus*, *Pseudomonas aeruginosa*, *Enterococcus faecalis*, and *Campylobacter jejuni*. An Au@PANI–IA–Fe_3_O_4_ composite has been studied against *Staphylococcus aureus* (Gram +ve) and *Escherichia coli* (Gram −ve) microbes in Mueller–Hinton agar (MHA) plates. It was observed that the composite shows a better zone of inhibition with increasing composite concentration against *Staphylococcus aureus* while the composite shows an insignificant inhibition zone for *E. coli* microbes. The antifungal property of the composite has been studied against *Candida albicans*. The result shows that the composite follows a trend of increased inhibition zone when the composite concentration is increased.^231^ The polyaniline NF/Ag nanoparticles have a synergetic effect against microbial action. The antimicrobial mechanism behind the Ag nanoparticles is partially known. The concentration of silver nanoparticles plays a crucial role in the case of Gram-negative microorganisms. The positively charged silver accumulates on the cell periphery, and this permeable particle causes the death of the microorganism. Selcuk Poyraz showed that PANI NF/Ag nanoparticles have more effective inhibition for Gram-negative *E. coli* than for Gram-positive *S. aureus* due to the difference in their peptidoglycan layouts because of the synergetic effect of Ag and polyaniline.^232^

A summary of studies on the use of conjugated polymers in biomedical applications is given in Table 4.

Summary of studies on the use of conjugated polymers in biomedical applications

**Table:** 

Polymer	Application	Type	Analyte	Reference
Polyaniline-supported Cu–CuO, core–shell	Sensing	Electrochemical	Glucose	233
Polypyrrole/CuS@SiO_2_		Voltametric	Glucose	234
Au@rGO/polyaniline		Electrochemical	Furazolidone and flutamide	235

**Table:** 

Polymer	Application	Type	Reference
Polycaprolactone–Polyaniline	Scaffolds	Bone tissue scaffold	236
Polypyrrole		Muscle tissue scaffolds	237
PVA/PEDOT		Nerve tissue scaffolds	238

**Table:** 

Polymer	Application	Irradiation	Temperature reached and cell line	Ref.
PLNP@PANI-GCS	Photothermal therapy	808 nm 1.5 W cm^−2^ 5 min	52.2 °C and MRSA	239
DOX–PPy@PNAs nanogel		808 nm 0.5 W cm^−2^ 5 min	47.9 °C and H22 tumor	240
Polypyrrole–rhodamine B		808 nm 0.26 W cm^−2^ 10 min	53 °C and HepG2 cells	241
S-rGO–Fe_3_O_4_–PANI		808 nm 2 W cm^−2^ 10 min	80 °C	228

### 6.5. Electrochemical gas sensors

The oldest version of an electrochemical sensor, during the 1950s, was useful for oxygen monitoring. Currently, well-designed electrochemical sensors have been successfully developed for the monitoring of combustible and toxic gases in space-related applications. Miniature forms of highly selective electrochemical sensors moved into the limelight with their ability to detect toxic gases in the PEL range. The appearances of various electrochemical sensors for gas sensing may be similar, but these sensors exhibit markedly different functions. Besides, the sensors show various performances attributed to their specificity, response time, sensitivity, and operational life. For example, a highly sensitive gas sensor with low concentration uses a hydrophobic porous membrane, and better sensitivity through sufficient signal production is possible due to less restricted capillary movement to allow the passage of more gaseous molecules. On the other hand, this design permits the escape of electrolyte water molecules to the outer environment. Besides, a highly sensitive electrochemical sensor should have a comparatively low operating life because of the evaporation of water molecules through the porous membrane. The selectivity of an electrochemical sensor for gas detection is based on sensor type, target gas, and the gas concentration the sensor has been developed to detect. Similarly, the chemical reactivity of a specific target gas depends on the composition of electrolyte and electrode material for the sensing process. Additionally, specific electrochemical sensors utilize electrical power to produce a response towards the target gas.^242^ The working of electrochemical sensors is based on the reaction with the target gas and generates an electrical signal proportional to the concentration of target gas. An electrochemical sensor is comprised of a working (sensing) electrode and a counter electrode separated by a thin electrolyte layer. Firstly, the gas passes through a tiny capillary kind of opening, followed by diffusion across a hydrophobic barrier and finally enters the electrode surface. This approach is explored to allow more gas molecules to reach the sensor to generate a better electrical signal while restricting the leakage of electrolyte out of the sensor electrode. The gas diffused across the barrier undergoes either an oxidation or a reduction mechanism on the surface of the sensor electrode. The current proportional to the concentration of gas molecules that flows between the anode and the cathode in an electrochemical cell is measured. This electrochemical sensor is usually described as a micro fuel cell or an amperometric gas sensor due to the current generated in this process.

In an electrochemical sensor, the potential at the sensing electrode is not constant due to the continuous electrochemical reaction on the surface of the sensor electrode. Hence it results in a decrease in the performance of electrode as time goes on. So, a reference electrode is used to improve the performance of the sensing electrode. A stable fixed potential is applied to the sensing electrode, and the reference electrode needs to maintain the value of the fixed potential at the sensing electrode without any current flow to or from the reference electrode. The value of fixed potential applied at the sensing electrode determines the specificity of the sensor to a target gas.

#### 6.5.1 Major components

Electrode: the electrode material is crucial for an electrochemical cell reaction. It is a catalytic material for performing the half-cell reaction over extended periods of time. The electrode is usually made of a material such as platinum or gold and is catalytically accelerated for an effective reaction with the target gas molecules. Three electrodes in an electrochemical cell can be composed of different materials according to the overall design of the electrochemical sensor. Electrolyte: this should be compatible with the electrode materials used in the sensor and allows an efficient transfer of ionic charges between the electrodes in order to promote the cell reaction. The signal deterioration will be faster if the electrolyte material evaporates too fast. Gas permeable membrane: This is used to protect the catalyst (sensing) electrode and to control the gas concentration entering at the electrode surface. These barriers are made of thin and low-porosity Teflon membranes, so the sensors are known as membrane clad sensors. In addition, there are sensing electrodes protected with highly-porous Teflon membranes, and the gas flow is controlled by a capillary movement. These sensors are known as capillary-type sensors. In some instances, the membranes are known to function as filters for removing undesirable particulates. The pore size of membranes should permit enough gas molecules to reach the sensing electrode and inhibit the leakage or evaporation of liquid electrolyte to the outer environment. Filter: filters have a different degrees of effectiveness. For example, a scrubber filter is used in front of the sensor to filter out undesirable gases. The most common charcoal filter is shown in the figure, which is used to filter out most gases except hydrogen and carbon monoxide. The proper selection of a suitable filter medium allows an electrochemical sensor to be more selective towards its target gases.

The proper working of the sensor is based on an adequate supply of oxygen molecules for the completion of the reaction; otherwise, a shortage of oxygen supply will shorten the lifespan of the sensors. It is essential to maintain the sensor at the same pressure; otherwise, changes in pressure may damage the electrochemical sensor. Electrochemical sensors are quite sensitive to temperature. If the temperature is above 25 °C, the sensitivity will be higher. If it is below 250 °C, the sensitivity will be lower. TMDs can be widely used in switching applications attributed to their intrinsic bandgap. Changes in TMD layers can be used for tuning of the bandgap between 1 and 2.05 eV due to quantum confinement. TMD materials with prominent mechanical, electrical, and chemical properties were also widely used for sensing applications. A single transition metal layer is comprised of an atomic plane sandwiched between the two atomic planes of chalcogenide atoms. Whereas in the multilayer form, layers are connected to each other by van der Waals forces. The transition metal atom is covalently attached to the chalcogenide atom. With a large interlayer surface area available for chemical adsorption, there is a notable improvement in electrical signals as it is able to detect the presence of chemical molecules even in low concentrations. TMDs like MoS_2_, MoSe_2_, VS_2_, WS_2_, WSe_2_, *etc.* are leading candidates in emerging gas sensing technologies due to their flexibility, transparency, and efficient gas adsorption. The main disadvantage of metal oxide and inorganic material gas sensors is their high operational temperature. A high operational temperature affects the durability of the sensors, which will be damaged within a short time. Conducting polymer-based sensors were a big breakthrough because of their fast response time, recovery time, and their low operational temperature. This allows us to work at room temperature, leading to a long lifespan.^243^

#### 6.5.2 Sensing mechanism

Charge transfer processes constitute the basis for gas sensing in graphene and similar 2D layered inorganic analogues, in which sensing materials function as either charge donor or acceptor. The exposure of the sensor material to different gases can generate a charge transfer between the adsorbed gas molecules and the sensing materials. This charge transfer involves different directions of transfer and charge quantities, which can change the resistance of the material. If the sensing material is re-exposed to gases, the resistance will return to its initial value and thereby lead to the desorption of gas molecules from the surface of the sensing material.^243^ The charge transfer mechanism between the conducting polymers and adsorbed gas molecules, such as NO_2_, O_2_, NH_3_, NO, H_2_O, or CO, has been investigated. As stated before, conducting polymers behave like an insulator or semiconductor in their pristine form but when doped with suitable materials, their conductivity changes to metallic. The backbone of conjugated polymers is constructed from conjugate bonds, consisting of both σ and π bonds. The σ bonds are responsible for the mechanical property of the polymers. Alternating π bonds and their delocalization and overlap with neighboring bonds form the electrical property.^244^ When the conjugated polymer is doped, it will generate charge carriers like polarons/bipolarons/solitons, *etc.* These charge carriers are delocalized along the chain to induce charge transport along the polymer backbone. On the other hand, interchain transport occurs by hopping or by charge tunneling. In the case of a p-type sensing material, an optimum amount of oxygen gets chemisorbed on the material surface, and this absorbed oxygen gets changed to single or double oxygen ions and is ionosorbed on the surface. The addition of oxygen molecules onto the substrate causes the removal of an electron from the sample surface. This causes an increase in hole density on the surface and causes a decrease in resistance. When reducing gases react with ionosorbed species, electrons are pumped into the conduction band of a p-type material. Hence, hole concentration decreases and causes an increase in the resistance. If oxidizing gases come into contact with ionosorbed species, this causes the depletion and removal of an electron from the valance band whereby hole density increases due to recombination, finally resulting in decrease in resistance. The working mechanism is the opposite in the case of n type materials. The dopant type classifies conducting polymers into p type or n type polymers (Table 5).

**Table:** Sensing response of reducing and oxidizing analyte

Sensing response	P type polymer sensor holes (h^+^)	N type polymer sensor electrons (e^−^)	Examples of analytes
Reducing analytes	Resistance increases	Resistance decreases	CO, NH_4_, CH_4_, H_2_, H_2_S, acetone, ethanol
Oxidizing analytes	Resistance decreases	Resistance increases	NO_*x*_, CO_2_, SO_2_, O_2_, O_3_

Among conducting polymers, polyaniline has been effectively utilized in the sensing field because of its easy synthesis, doping and de-doping behavior, ease of deposition on substrates and reactivity towards gases. Doping will elevate the conductivity range with high reversibility; which is why it is widely using for the sensing of toxic gases like CO, NO_2_, *etc.* The secondary components in a conducting polymer composite can be metallic, bimetallic, transition metal chalcogenides, carbonaceous materials, polymers, *etc.* These secondary nanoparticles are stabilized with a polymer matrix by a weak coulombic force or by van der Waals force. The introduction of secondary particles modifies the physical and chemical properties of the conducting polymers and this tendency has been utilized for various applications. Kumar *et al.* studied the sensing activity of PANI/Au NS as an NH_3_ gas sensor, and they observed that Au NS act as a catalyst and the composite gives a better response time of 15 s, which is quicker than that of pure Au NS. The sensing response increases by 52% when a composite is made with polyaniline.^245^ The use of graphene is limited in digital circuits on flexible and wearable sensing devices due to the absence of a bandgap. Thus, the semiconducting properties of pristine graphene remain a hindrance for gas sensing applications. The semiconducting properties of sensing materials are a crucial factor for tuning the sensing characteristics of a gas sensor. A composite with a conducting polymer overcomes the above limitations of polyaniline. Wu *et al.* fabricated a graphene/polyaniline sensor for the detection of NH_3_ gas at different concentrations. From their studies, they observed that the sensor acts with conductometric behavior and the resistance of the sensor increases when exposed to analyte gas. The sensor retaliation value varies with analyte concentration. The composite shows sensor responses of 3.65 and 11.33% at concentrations of 20 and 100 ppm of NH_3_ analyte. The response time and recovery time of the composite at 100 ppm concentration were noted to have values of 50 s and 23 s.^246^ Hydrogen gas is colorless, odorless, and tasteless, and shows explosive behavior above a critical concentration range. Hydrogen gas is used as a fuel in various industries. Due to its high permeability, it can cause explosions. The precise monitoring of hydrogen gas is critical due to the above problems. An exact and precise sensing mechanism is needed for the detection of hydrogen gas leakages. Polyaniline based sensors are used for the detection of hydrogen gas effectively and precisely. Sharma *et al.* developed Al-SnO_2_/PANI nanofibers *via* an electrospinning technique, and their sensing activity was studied thoroughly. A 1% Al-SnO_2_/PANI sensor shows a high sensitivity range of ∼275% to H_2_ gas at 1000 ppm. The sensor has a rapid sensing and recovery time of 2 s at a concentration range of 1000 ppm at 40 °C.^247^ Wang *et al.* fabricated a polypyrrole/Fe_2_O_3_ nanocomposite for the detection of NO_2_ gas. Fe_2_O_3_ present in the composite enhances the response due to the p–n heterojunction. This sensor shows precise sensing activity even at a lower concentration of 0.1 ppm and the sensor shows a linear response in a concentration range of 0.1–10 ppm. The composite shows a good response of 220.7%, which is higher than in previous available reports.^248^ Kwon *et al.* developed a PEDOT:PSS/PVP composite by an electrospinning technology. The sensor was effectively used for the detection of various vapors like ethanol, methanol, tetrahydrofuran (THF), and acetone. The sensor has improved sensing actions like excellent reversibility, reproducibility, response, and recovery time. The composite maintains almost the same response and recovery time with different cycles but the sensor resistance decreases when exposed to polar protonic solvents.^249^

### 6.6. Conducting polymer hydrogels (CPH) for bioelectronics

Conducting polymer hydrogels are a combination of conducting polymer and hydrogels in order to achieve some novel properties that are not available for conventional materials. The conducting polymer hydrogels consist of conducting polymers like polypyrrole, polyaniline, or polythiophene crosslinked covalently or physically with hydrophilic networks.^250,251^ The hydrogel morphology is easily tunable and it has different applications, including sensing, bioelectronics, OECT, tissue engineering and energy storage applications.^252–254^ The main advantages of hydrogels are their tunable chemical and physical properties when subjected to doping engineering. In the case of polypyrrole hydrogels, their morphology varies from nanofibres to 3D porous structures in accordance with the doping and nature of the dopant. CPH has high mechanical, electrical, self-healing and biocompatibility properties.^255^ They are widely applied in bioelectronics and tissue engineering. Conducting polymers alone are not sufficient for certain applications due to their lack of long-term stability. Some research groups have proposed the use of conducting hydrogels instead of conducting polymers to achieve long-term stability. CPH is fabricated using different techniques like free radicals, click chemistry, condensation and Michael-type addition polymerization. The chemical techniques are studied widely for biomedical applications.

The invention of bioelectricity led to research into understanding the communication between biology and electronics. The electronic response of the nervous system enables the muscular movements, memory and reasoning ability of humans. Generally, bioelectronic interfaces are electrodes which interact with biological tissues. They collect bioelectronic signals and enable personalized self-monitoring of tissues.^256,257^ Bioelectronics are widely used in various applications, including electronic skin, the detection of brain activity, soft robotics and wearable and flexible implantable devices.^258^ Before the invention of CPH, inorganic materials were widely used for the fabrication of bioelectronic devices. Its replacement is proposed due to its unreliability in signal collection, cytotoxicity and inflammatory response by the body. Bioelectronic devices need to maintain high conductivity and high mechanical properties in accordance with the movement of human body parts, which is very difficult. Transistors like organic electrochemical transistors (OECTs)^259^ and electrolyte-gated organic field effect transistors are generally used for bioelectronic sensing. OECTs have aroused great research and industrial interest due to their high transconductance, biocompatibility and processability.^260^ An OECT is constructed from a semiconductor/organic film in contact with two electrodes, a drain and a source, an electrode (gate) immersed in a electrolyte. The organic film enables the movement of charge and ions from source to drain. The channels of the OECT are fabricated using PEDOT:PSS, a polypyrrole polymer doped with p-type dopants.^261^

Bioelectronic devices are the basic unit for the fabrication of wearable/implantation devices for tissue response monitoring. Conducting polymer hydrogels are used to fabricate pressure and strain sensors due to their ability to decode mechanical signals to electrical signals. Flexible electrodes made up of conducting polymer hydrogels are widely used to monitor electrophysiological signals, including ECG,^262^ electromyography and electroencephalography signals.^263^ Generally, electrophysiological signals are very weak. The main challenge for device fabrication is to enhance the conductivity and signal-to-noise ratio. Skin adhesion, long-term stability and biocompatibility are also taken into consideration. PAA/PEDOT hydrogels have been designed to detect different electrophysiological signals. Polyaniline based hydrogels are used for real-time glucose and lactate monitoring.^264^ OECT is widely used for monitoring electrophysiological signals, including ECG, electromyography, and electroencephalography.

The conductivity of hydrogels can be increased by adding conductive fillers. The conductivity of a polyaniline/phytic acid hydrogel was recorded as 0.21 S cm^−1^. After the incorporation of MWCNT, the conductivity increased to 1.54 S cm^−1^.^265^ Similarly, an enhancement in the specific capacitance of various conducting polymer hydrogels was noted with the introduction of foreign conductive fillers. If the filler loading is beyond a particular amount, the hydrogels show a phase separation between polymer matrix and filler, causing low mechanical stretchability. So, it is very difficult to balance the mechanical stretchability and conductivity. There have been a lot of efforts to maintain stretchability without losing conductivity. The stretchability of a conducting PEDOT:PSS polymer was enhanced by 35% by structural modification in the interconnected fibrillar structure through solvent annealing. The stretchability was also improved by the introduction of plasticizers into the polymer matrix. The conductivity is same with respect to pristine form even after application of a 100% strain condition.^266^

## 7. Conclusion and future directions

To date, there have been a lot of studies done on the fabrication of conducting polymers. The main aim of this review article has been to explore current trends in the synthesis, properties, and applications of conducting polymers. Conducting polymers and their composites are always in the limelight because of their metallic conductivity when doped and excellent physical properties. From literature reviews, it was observed that conducting polymers in their pristine form behave as an insulator or a semiconductor. They show metallic conductivity only when doped with a suitable dopant or when made into a composite with foreign materials. The physical and chemical properties of conducting polymers also depend on morphology; different morphology gives distinguishable properties. Understanding the basic properties is essential for the design of conducting polymers for various applications. The complex structure of conducting polymers and their derivatives poses a difficulty for theoretical modelling; therefore, an understanding of the origin of conductivity and the doping mechanism is essential. Charge carriers are developed on a polymer backbone either when added to or extracted from the delocalized π bond. Various parameters like temperature, dopants, and structural properties have an effect on transport properties.

Conducting polymers are widely used as supercapacitor electrode materials because of their metallic conductivity, flexibility, processability, and ease of fabrication. Most conducting polymers exhibit high specific capacitance, and they deliver energy rapidly. The main disadvantage of conducting polymer-based supercapacitors is their cycle life; symmetric conducting polymer supercapacitors have a lower cycle life than carbonaceous material-based supercapacitors. From the literature, we can observe that the cycle life problem can be overcome by irradiation, sonication during synthesis or compositing with carbonaceous or non-carbonaceous materials. In the case of an asymmetric configuration, conducting polymers and their derivatives as positive electrodes and carbonaceous material as negative electrodes provide a higher cycle life and specific capacitance. From the literature, we can find that stability and cycle life can be improved by incorporating metal oxides, transition metals, transition metal dichalcogenides, and carbonaceous materials. From these studies, we can understand that future developments and research should be carried out into a hybrid conducting polymer composite that will prove effective in achieving better supercapacitor performance.

Due to the environmental and health issues of chromate conversion coatings, researchers have put effort into finding an alternative. Conducting polymers have been widely used for corrosion inhibition of metals and their alloys during the last few decades. They are also used as pigments for organic coatings. Also, multilayers of conducting polymers and their composites act as corrosion inhibitors. The dopants incorporated into the polymer backbone release necessary inhibitors during reduction. Conducting polymer films electrochemically synthesized in the presence of suitable electrolytes act as efficient inhibitors against corrosion, and the film thickness can be modified by controlling the electrochemical parameters. Organic coatings like epoxy, acrylic, polyurethane, *etc.* added to the conducting polymers improve their corrosion inhibition property. Morphology plays a vital role in corrosion inhibition. The controlled release of inhibitors upon reduction is the main advantage of using polymer coatings. Also, they generate a metal oxide layer beneath the topcoat in aggressive environments, and these metal oxide layers prevent corrosion effectively and efficiently. From an analysis of recent papers, future developments should focus on conducting polymer incorporated organic coatings for better corrosion inhibition activity. The durability of coatings increases with compositing with organic coatings.

The performance of conducting polymer-based photocatalysts depends upon the rate of photoelectron transfer to the CB of particles incorporated on the polymer backbone like metal oxide, TiO_2_, *etc.* In the literature, it is shown that high photocatalytic activity is achieved only when electron–hole combination is minimized. From a literature review, it is observed that the performance of conducting polymer sensitized TiO_2_ photocatalysts depends upon a lot of factors like structural arrangements. The covalent bond enhances electron coupling, and excited electrons in the conducting polymer can be injected to the CB of TiO_2_. Until now, reports have not been available on conducting polymer composite based photocatalysts, so future development should focus on different composites of conducting polymers.

Conducting polymers are new and promising materials in the field of biomedical engineering but have not been wholly explored until now. There are some limitations to conducting polymers for biomedical applications due to their cytotoxic nature and differences in in vivo and *in vitro* studies. Generally, physical properties and biocompatibility issues are the main problems for conducting polymers. Most of the biocompatibility issues of conducting polymers can be overcome by compositing with Ag or with biodegradable polymers. From the literature, we can observe that polyaniline based composites have good photothermal conversion efficiency and they have a lot of advantages over other conducting polymers. They have high water solubility, high NR absorbance, high therapeutic action, and minimum cytotoxicity. There has been less research on conducting polymers, and future research should focus on different conducting polymer composites.

Among conducting polymers, polyaniline and polypyrrole have been effectively used for electrochemical sensing applications. Nanostructures of conducting polymers have great prospects in sensing action because of their high surface area or high surface to volume ratio for the diffusion of analyte gas molecules into and out of the polymer matrix, compared to their bulk form. The conducting polymer morphology and film thickness play an essential role in the sensing action because of inter-domain spacing, and this will reduce the interaction of analyte gas with the polymer. Polyaniline has better sensing behavior than the others due to its reversible doping mechanism. Hybrid conducting polymers overcome the drawback of selectivity and the high working temperature problem of metal oxide chemiresistors. Polyaniline and polypyrrole conducting polymers, and their combinations, should be explored more for the detection of oxidizing and reducing gases. Future developments should focus on other conducting polymers.

## Conflicts of interest

The authors declare no conflict of interest.

## Supplementary Material
