# Correction: Conducting polymers: a comprehensive review on recent advances in synthesis, properties and applications

**DOI:** 10.1039/d4ra90058h

**Published:** 2024-05-30

**Authors:** Namsheer K, Chandra Sekhar Rout

**Affiliations:** a Centre for Nano and Material Sciences, Jain University, Jain Global Campus Jakkasandra, Ramanagaram Bangalore 562112 India r.chandrasekhar@jainuniversity.ac.in

## Abstract

Correction for ‘Conducting polymers: a comprehensive review on recent advances in synthesis, properties and applications’ by Namsheer K *et al.*, *RSC Adv.*, 2021, **11**, 5659–5697, https://doi.org/10.1039/D0RA07800J.

In the original manuscript, the authors regret errors in the schemes, charts and figures as detailed below.

In Chart 2, the structures of emeraldine and pernigraniline contain multiple carbons drawn incorrectly with 5 bonds, and the structure of pernigraniline is incorrectly labelled as being fully reduced rather than oxidised. The correct structures and labels are indicated herein.

**Chart 1:**
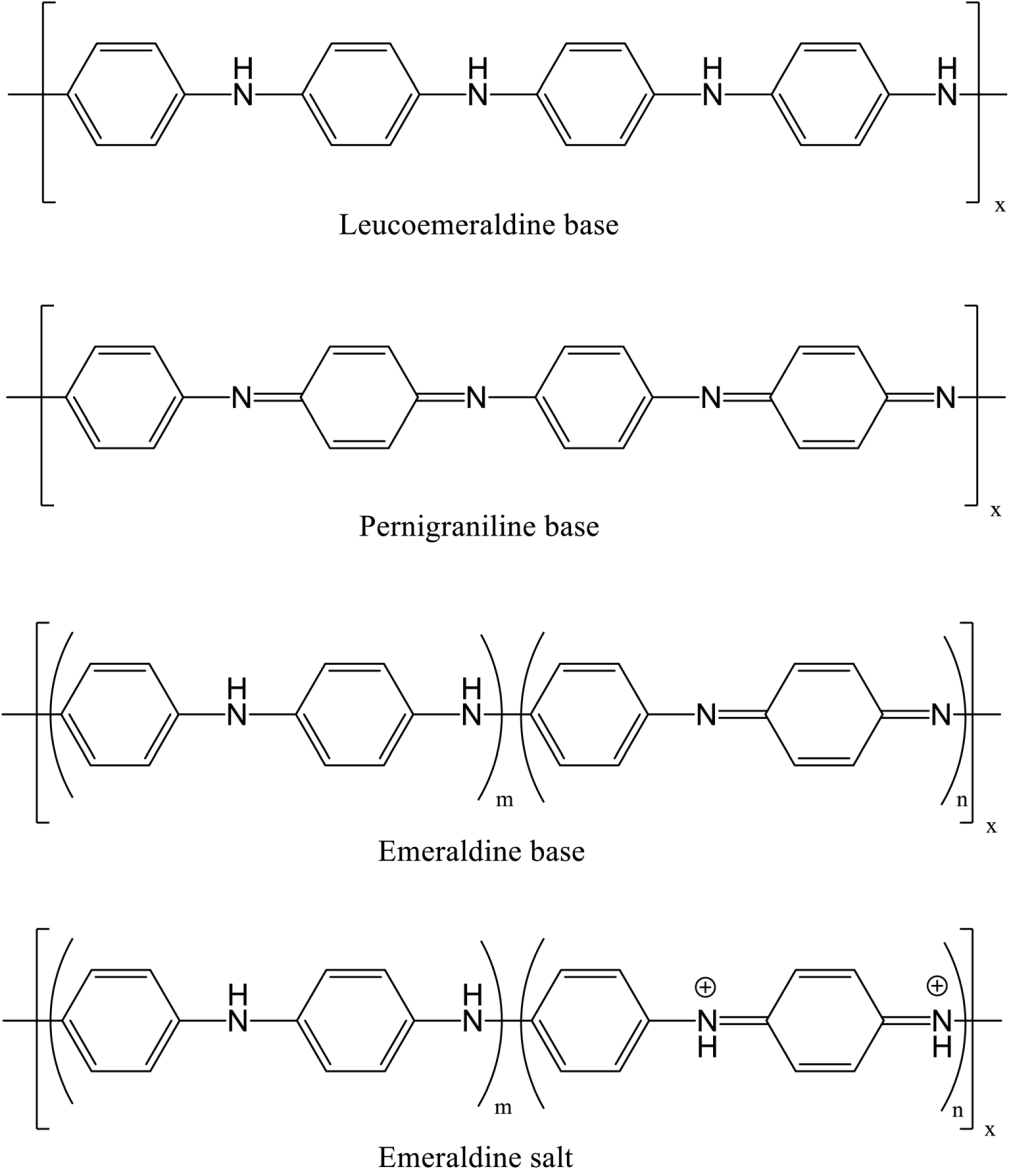
Structural illustration of different forms of polyaniline.

In Scheme 8, the chlorine atoms are written as ‘cl’ rather than ‘Cl’. A corrected Scheme 8 is shown herein.

**Scheme 1:**
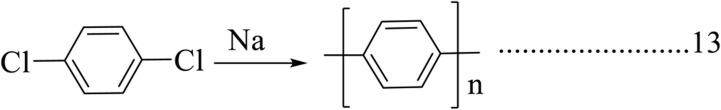
Synthesis of poly(*p*-phenylene)s using a Wurtz–Fittig reaction.

In Scheme 10, P + Ph_3_ was drawn incorrectly, and should read as P^+^Ph_3_.

**Scheme 2:**
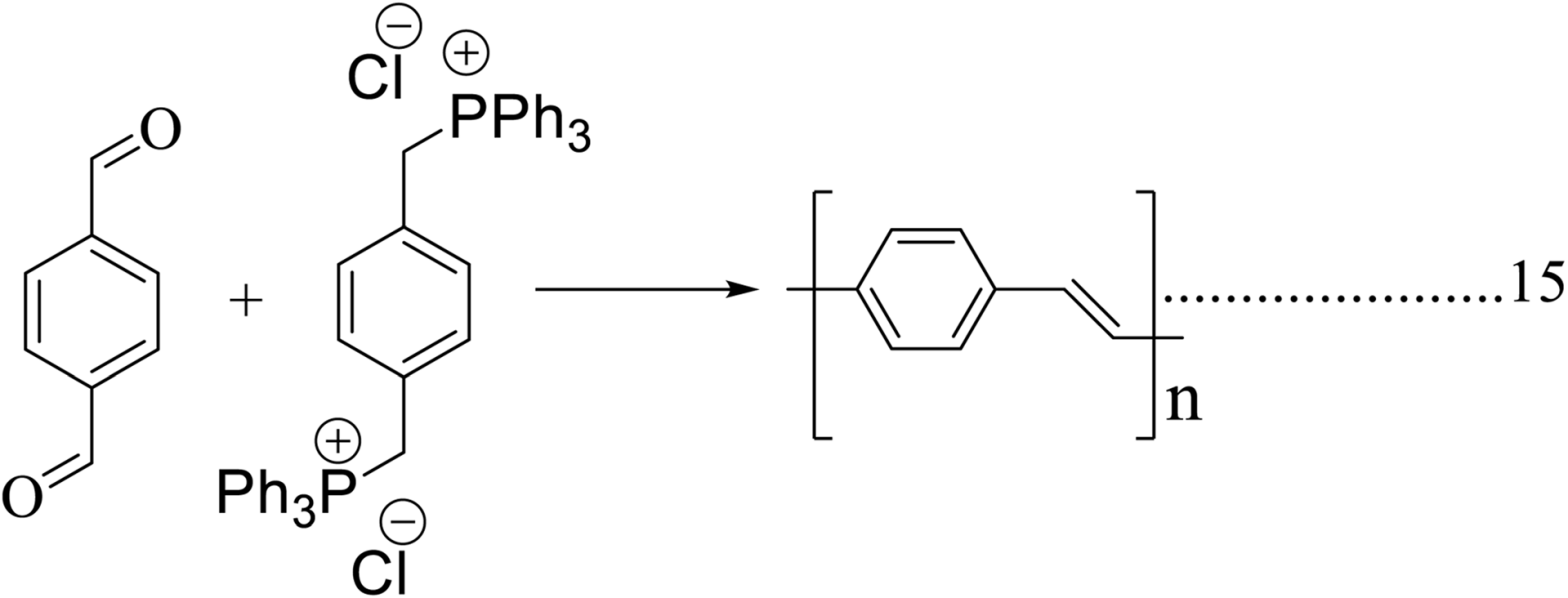
Synthesis of poly(*p*-phenylene vinylene) by a Wittig coupling reaction.

Finally, Fig. 7 contained formatting errors. The correct version of Fig. 7 can be viewed herein.

**Fig. 1:**
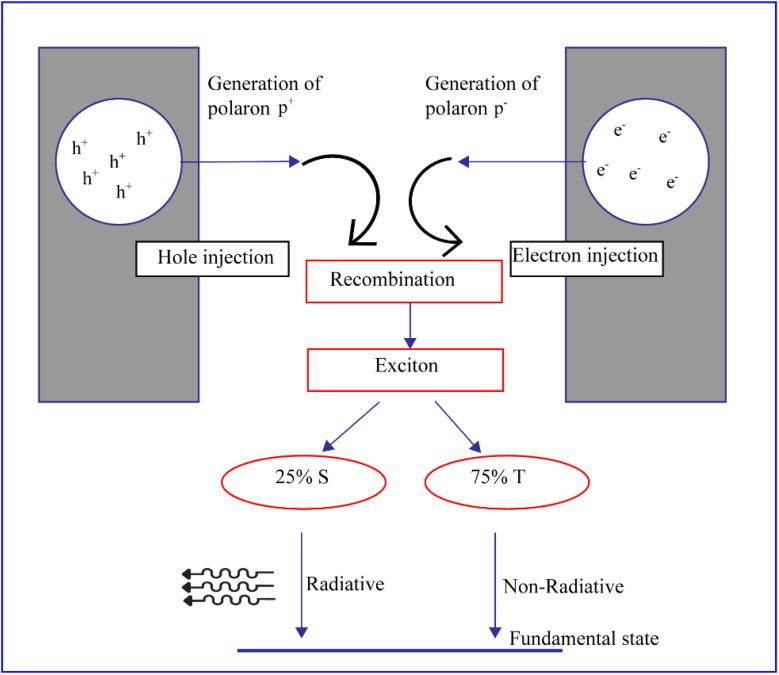
Schematic illustration of electroluminescent mechanism of conducting polymer diodes.

These revisions do not affect the conclusions of the article.

The Royal Society of Chemistry apologises for these errors and any consequent inconvenience to authors and readers.

## Supplementary Material

